# COVID-19 epidemiological, sociological and anthropological investigation: study protocol for a multidisciplinary mixed methods research in Burkina Faso

**DOI:** 10.1186/s12879-021-06543-4

**Published:** 2021-09-03

**Authors:** Isidore Tiandiogo Traoré, Samiratou Ouedraogo, Dramane Kania, Firmin Nongodo Kaboré, Blahima Konaté, Rachel Médah, Hermann Badolo, Nathalie de Rekeneire, Ariane Mamguem Kamga, Armel Poda, Arnaud Eric Diendere, Boukary Ouédraogo, Esperance Ouédraogo, Oumar Billa, Halidou Tinto, Tienhan Sandrine Dabakuyo-Yonli

**Affiliations:** 1 Centre MURAZ, Institut National de Santé Publique, Bobo-Dioulasso, Burkina Faso; 2 Institut Supérieur Des Sciences de la Santé, Université Nazi Boni, Bobo-Dioulasso, Burkina Faso; 3 Institut National de Santé Publique du Québec, Montréal, QC Canada; 4 The Department of Epidemiology, Biostatistics and Occupational Health, Faculty of Medicine, McGill University, Montreal, QC Canada; 5 Observatoire National de la Santé de la Population, Institut National de Santé Publique, Ouagadougou, Burkina Faso; 6 Département de Socio-Économie et d’Anthropologie du Développement, Institut des Sciences des Sociétés - Centre National de la Recherche Scientifique et Technologique, Ouagadougou, Burkina Faso; 7 Expertise France, Paris, France; 8 Epidemiology and Quality of Life Research Unit, INSERM U1231, Georges Francois Leclerc Centre – UNICANCER, Dijon, France; 9 Service des Maladies Infectieuses, Centre Hospitalier Universitaire Sourô Sanou, Bobo Dioulasso, Burkina Faso; 10 Centre Hospitalier Universitaire de Bogodogo, Ouagadougou, Burkina Faso; 11 Direction des Systèmes d’information en Santé, ministère de la santé, Ouagadougou, Burkina Faso; 12 Département de Médicine, pharmacopée traditionnelle et pharmacie, Institut de Recherche en Sciences de la Santé - Centre National de la Recherche Scientifique et Technologique, Ouagadougou, Burkina Faso; 13 Institut de Recherche en Sciences de la Santé, Centre National de la Recherche Scientifique et Technologique, Ouagadougou, Burkina Faso

**Keywords:** SARS-CoV-2, COVID-19, Sero-epidemiology, Clinical epidemiology, Predictive score, Socio-anthropology

## Abstract

**Background:**

The world has high hopes of vaccination against COVID-19 to protect the population, boost economies and return to normal life. Vaccination programmes are being rolled out in high income countries, but the pandemic continues to progress in many low-and middle-income countries (LMICs) despite implementation of strict hygiene measures. We aim to present a comprehensive research protocol that will generate epidemiological, sociological and anthropological data about the COVID-19 epidemic in Burkina Faso, a landlocked country in West Africa with scarce resources.

**Methods:**

We will perform a multidisciplinary research using mixed methods in the two main cities in Burkina Faso (Ouagadougou and Bobo-Dioulasso). Data will be collected in the general population and in COVID-19 patients, caregivers and health care professionals in reference care centers: (i) to determine cumulative incidence of SARS-CoV-2 infection in the Burkinabe population using blood samples collected from randomly selected households according to the WHO-recommended protocol; (ii) develop a score to predict severe complications of COVID-19 in persons infected with SARS-CoV-2 using retrospective and prospective data; (iii) perform semi-structured interviews and direct observation on site, to describe and analyze the healthcare pathways and experiences of patients with COVID-19 attending reference care centers, and to identify the perceptions, acceptability and application of preventive strategies among the population.

**Discussion:**

This study will generate comprehensive data that will contribute to improving COVID-19 response strategies in Burkina Faso. The lessons learned from the management of this epidemic may serve as examples to the country authorities to better design preventive strategies in the case of future epidemics or pandemics.

The protocol was approved by the Ministry for Health (N° 2020-00952/MS/CAB/INSP/CM) and the Health Research Ethics Committee in Burkina Faso (N° 2020-8-140).

**Supplementary Information:**

The online version contains supplementary material available at 10.1186/s12879-021-06543-4.

## Background

In late 2019, clusters of infection with a novel coronavirus began to appear in China, often progressing to fatal pneumonia [1, 2]. The infections were due to a highly contagious virus identified on 9 January 2020, called Severe Acute Respiratory Syndrome Coronavirus 2 (SARS-CoV-2), and the associated disease was termed COVID-19 (Coronavirus Disease 2019) [3]. Within a few short weeks, the infection went from a localized epidemic to a worldwide pandemic [4] with more than a million persons contaminated in over 209 countries, and more than 80,000 deaths by 9 April 2020, i.e., only 100 days after notification of the first case [5]. In April 2020, Burkina Faso was experiencing a rapid increase in the number of cases and deaths and ranked as the fifth most affected country in Africa [6, 7], even though the first cases had only appeared one month earlier, and several weeks after the first cases reported in Europe. Epidemic cycles with emergent or re-emergent diseases, combined with endemic diseases, put considerable strain on the health system of Burkina Faso.

Research on COVID-19 has been intensifying around the world [8–12], with numerous ongoing randomized trials including the “Discovery” trial [13] initiated by the European Union, or the “Solidarity” trial, initiated by the WHO [14]. Preliminary results from this latter trial indicate that treatment regimens using remdesivir, hydroxychloroquine, lopinavir/ritonavir, and interferon unfortunately do not appear to be effective in terms of 28-day mortality or the course of disease in hospitalized patients [14]. In Burkina Faso, therapeutic trials are also ongoing [15], notably the CHLORAZ study, which is an observational study comparing the effect of a combination of hydroxychloroquine plus azithromycin (HCQ-AZ) versus the combination of chloroquine phosphate plus azithromycin (CQ-AZ) for the treatment of COVID-19.

However, the greatest hopes for vanquishing this pandemic lie in the development of vaccines, and several countries in Europe and North America have already approved vaccines and commenced population-level immunization. Vaccines already approved and in use include the Pfizer-BioNTech [16], Astra Zeneca [17], Janssen COVID-19 [18], and Moderna mRNA-1273 [19] vaccines. However, with limited financial and infrastructural resources, it remains uncertain whether countries like Burkina Faso will be able to procure and distribute COVID-19 vaccines for the entire population. Therefore, they must focus more than ever on non-pharmacological measures, namely handwashing, masks, physical distancing, curfews, and closure of schools and businesses. Yet, these measures are sometimes difficult to implement and require socio-anthropological information to better orient prevention strategies. In this paper, we aim to present a comprehensive research protocol that will generate epidemiological, sociological, and anthropological data about the COVID-19 epidemic in Burkina Faso, a country where resources are scarce.

Since March 2020, Burkina Faso has been implementing a strategic plan to fight against the epidemic that consists primarily in management of persons with suspected or confirmed SARS-CoV-2 infection with a view to limiting the spread of disease. The plan has contributed to containing the epidemic thus far, but numerous aspects of patient management and prevention of this novel disease remain unexplored and could help to guide public policy and enable the health system to deal more effectively with increases in cases.

Firstly, current techniques for COVID-19 diagnosis are expensive (e.g., PCR, for detection of genetic material from the virus) [20, 21], limiting the number of analyses that can be performed. This makes it difficult, not to say impossible to estimate the actual number of persons infected by the virus in the general population of Burkina Faso. Furthermore, the full spectrum of disease, and the relative proportions of benign or asymptomatic cases not requiring medical care, remain unclear [22]. As in many low- and middle-income countries, in Burkina Faso, the management of the pandemic has concentrated primarily on patients presenting with severe forms of disease. Although recommended by the WHO, few countries have assessed seroprevalence at the population level. According to available studies, seroprevalence for SARS-CoV-2 infection among the general population remains low (below 10%) [22] and reports mainly stem from western countries. In this context, in Burkina Faso, and in Africa in general, there remains some uncertainty as to the principal epidemiological and serological features of this novel pathogen, in particular its capacity to spread among the population, and its virulence. Yet, surveillance of seropositivity for IgG and IgM antibodies [23, 24] would make it possible to detect the spread of infection and estimate the cumulative incidence in the population [22]. It would also give insights during the pandemic into the proportion of the population already exposed to the virus, and thus, potentially immunized.

To the best of our knowledge, scores to predict patients at risk of severe forms of disease, or death, from among those with COVID-19 are not currently available in Burkina Faso or other countries with limited resources. Such scores would make it possible to focus preventive strategies and available resources on this high-risk sector of the population. Such scores might also contribute to reducing the costs associated with management of COVID-19 patients in a country where resources are scarce. To date, few studies have investigated the creation of a score or algorithm for predicting the occurrence of severe complications in patients with COVID-19 [25, 26], especially in African populations where the incidence rate and mortality are lower than the alarmist forecasts of international health authorities at the beginning of the pandemic.

Finally, preventive strategies against COVID-19 were developed and rapidly disseminated to the Burkinabe population by the media, associations, non-governmental organizations, local elected officials, religious and cultural leaders, and healthcare professionals, without giving a second thought to the acceptability of the measures being promoted for the population. However, the literature as well as media reports indicate that during other epidemics, notably Ebola [27–30], the implementation of preventive strategies (including raising awareness among the population, institutional isolation of infected individuals, collective management of cadavers by specialized healthcare teams) led to misunderstanding and conflictual interpretations between national or international scientific experts, and local populations. This breakdown in communication led to increased mortality, underlining the importance of evaluating the acceptability of preventive strategies for the population in the specific context of Burkina Faso. In addition, several specialists have questioned the feasibility of social distancing and isolation of suspected infected individuals in African populations where the community-based culture prevails. There are also unanswered questions regarding the pathways of care, patient management, and patients’ experiences. The specific objectives of this project comprise three phases, namely:

- To determine cumulative incidence (i.e., seroprevalence) of SARS-CoV-2 infection in the Burkinabe population (phase 1: sero-epidemiological study);
- To identify the predictors of the occurrence of severe complications and to identify the prognostic factors for mortality (phase 2: clinical epidemiology);
- To analyze the healthcare pathways and patient experiences of management of COVID-19 in healthcare centers dedicated to SARS-CoV-2 infected patients, and to explore the perceptions, acceptability, and application of preventive strategies among the population (phase 3: socio-anthropological study).

Embracing all these problems, we developed the present project, whose overarching aim is to generate novel, pragmatic epidemiological and socio-anthropological data using robust scientific methods, to support Burkina Faso and guide policy in its fight against the COVID-19 pandemic.

To the best of our knowledge, this will be one of the first studies of this type to be performed in Sub-Saharan Africa since the emergence of COVID-19.

## Methods

### The design and setting of the study

**Fig. 1:**
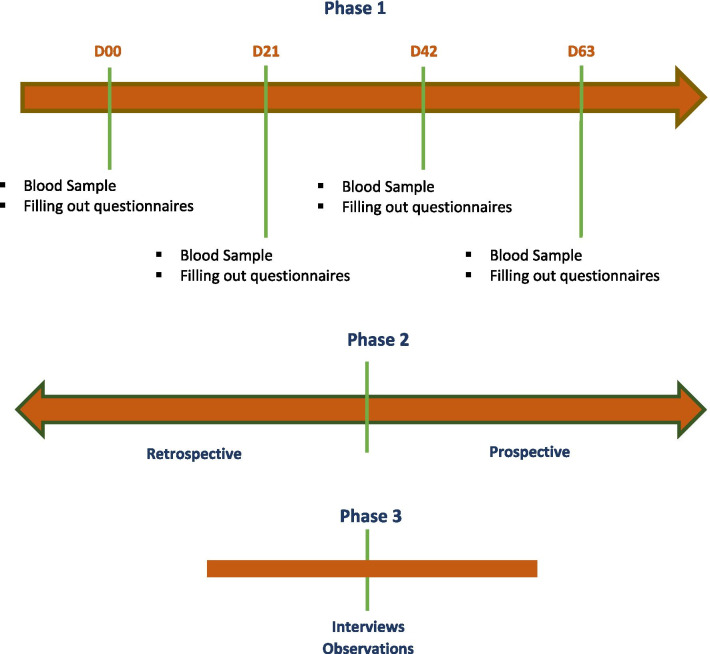
The three phases of a multidisciplinary research on COVID-19 in Burkina Faso

This will be a multidisciplinary study using mixed methods (i.e., combining quantitative and qualitative methods), in three distinct parts, detailed in Fig. 1. Each part of the study is presented separately.

It will be conducted in 2021 in Ouagadougou and Bobo Dioulasso, the two main cities of Burkina Faso, a landlocked country in West Africa.

## Phase 1: Sero-epidemiological study

For the investigation of seroprevalence, the protocol recommended by the WHO for sero-epidemiological studies in the general population will be followed [7].

### The characteristics of participants

A longitudinal cohort will be constituted from among the general population in the cities of Ouagadougou and Bobo-Dioulasso (Burkina Faso), which are the hardest hit by the pandemic. Households and persons will be randomly selected, with stratification by age group (60 and over, 19–59 years old, teenagers aged 15–18 and those aged 10–14 years) and gender (male, female) and included in the study. Random selection of the households will be performed based on census units of the national statistics institute of Burkina Faso. A household is considered as at least two persons living in the same dwelling, to consider so-called “celibatoriums”, which are grouped premises comprising several individual lodgings. In each household, all the members of the household will be invited to participate in the study, to collect data at days 0, 21, 42 and 63, to enable analysis by subgroup (age and gender).

### Inclusion criteria

Published statistics to date regarding COVID-19 in Burkina Faso indicate that there are very few cases in persons under 10 years of age.

The inclusion criteria for the study are as follows:

- Age 10 years or older.
- Resident in a census unit of the cities of Ouagadougou or Bobo-Dioulasso that is selected for participation in the study, based on data from the national statistics institute of Burkina Faso.
- Provide written informed consent.

### Non-inclusion criteria

- Persons aged 10 years or over from within the selected zones in Ouagadougou and Bobo-Dioulasso who refuse to participate in the study;
- Persons under legal guardianship or tutorship or other form of legal protection.

### Study endpoints

The primary endpoint will be the proportion of the population with specific antibodies against SARS-CoV-2.

Secondary endpoints will be as follows:

- The proportion of the population with specific antibodies against SARS-CoV-2 in each subgroup of age (60 and over, 19–59 years old, teenagers aged 15–18 and those aged 10–14 years) and by gender (males, females).
- The proportion of asymptomatic infections (presence of antibodies in patients with no clinical signs of SARS-CoV-2 infection) in the population, in each subgroup of age (60 and over, 19–59 years old, teenagers aged 15–18 and those aged 10–14 years) and by gender (males, females).

### Sample size calculation

Using the sample size calculator available on Openepi [31] (http://www.openepi.com/SampleSize/SSPropor.htm), with an expected SARS-CoV-2 seroprevalence of 10%, an accuracy of ± 3%, an alpha risk = 5% and no design effect (Deff = 1), we need at least 384 persons in each gender and age stratum at the final round (fourth timepoint). This number has been rounded to 452 to account for approximately 85% usable data.

Therefore, considering a non-response rate of 70% at inclusion and 30% between the data collection rounds (one to four), Table 1 (below) gives the number of subjects required per age and gender group per visit. At the final round (fourth timepoint), there should be 452 for each stratum. The details of the sample size calculation are presented in Table 2.

**Table 1:** Distribution of the city zones for the socio-anthropological study

	Bobo Dioulasso	Ouagadougou
Residential/central	1	1
Residential /peripheral	1	1
Poorer/central	1	1
Poorer/ peripheral	1	1
Total	4	4

**Table 2:** The study sample calculation strategy

Age and gender groups	Average number of people per household	Average number of households per EA* in the study cities	Number of EA needed for the study	Estimated number of people in the selected EA	Estimated number of people to contact	Estimated number of people to select in each EA	Estimated number of people needed after round 1	Estimated number of people needed after round 2	Estimated number of people needed after round 3	Estimated number of people needed after round 4
Men and women aged 60 and over	0.15	220	160	5280	4393	28	1318	922	646	452
Men aged 19 to 59	1.26	220	160	44,352	4393	28	1318	922	646	452
Women aged 19 to 59	1.10	220	160	38,720	4393	28	1318	922	646	452
Teenagers aged 15 to 18	0.51	220	160	17,952	2152	14	0	0	646	452
Teenagers aged 10 to 14	0.61	220	160	21,472	2152	14	0	0	646	452
Total				127,776	17,483	112	3953	2767	3229	2260

EA* Enumeration Area

To avoid the design effect (Deff = 1) in the calculation of the sample size, the sampling method will be two-stage random sampling with a probability proportional to their size in the resident population according to the data from the National Institute of Statistics and Demography survey (INSD).

To contact the 17,483 people for inclusion we will randomly choose 160 Enumeration Areas (EA) in Ouagadougou and Bobo-Dioulasso. In the two cities together 160 EA out of 1728 will be selected with a probability proportional to their size.

After selection of the EAs, the selection of individuals (second degree), will be done as follows:

- Complete a census and numbering of all concessions (can include several households), all households and target persons (men and women aged 60 and over, women aged 19–59, men aged 19–59, teenagers aged 15–18 and children aged 10–14 years),
- Number (from 1 to n) all the targeted residents by category,
- From each category, 28 residents will then be selected among adults and 14 teenagers from every EA.

If a selected participant is unavailable or refuses to participate in the study, he/she will be replaced with an individual randomly chosen from the same age and gender group in the same EA and in the same city. If the number of participants to select cannot be reached in an EA, it will be completed with the subsequent individuals in the randomization list. This strategy allows implicit stratification and is easy to perform. It simply requires that the sampling frame be sorted geographically before sampling systematically, with a probability to be selected proportional to the size. It also automatically provides a sample that is proportionally distributed among geographic subdivisions in the study area. Furthermore, it will make it easier to find the respondents in the field, since the interviewers will already be in touch with them through the census. Sample collection of teenagers will start at round three, enabling field investigators to obtain parental consent for their children to participate in the study during the first two rounds of data collection.

### Data collection and management

Data collection in the general population will take place at four timepoints, each 21 ± 3 days apart. Blood samples will be drawn by trained and qualified personnel. In each household, the members of the household who accepted to participate will be invited to sign the informed consent form, and complete a questionnaire (Additional file 1) with socio-demographic data, comorbidities, symptoms related to SARS-CoV-2 infection and exposure, namely:

- Socio-demographic characteristics: age, profession, marital status, level of education, socio-professional category;
- Prior medical history:
- History of cardiovascular and thrombo-embolic disease: arterial hypertension, rhythm disturbances, cardiomyopathy, deep venous thrombosis, pulmonary embolism;
- History of metabolic diseases: diabetes mellitus, kidney failure;
- History of pulmonary disease: tuberculosis, asthma, chronic obstructive pulmonary disease (COPD);
- Other relevant medical history.
- Lifestyle habits: tobacco and alcohol consumption;
- Clinical signs of COVID-19: fever (temperature ≥ 38 °C), cough, dyspnea, etc.;
- Exposure to COVID-19: any contact with a confirmed case of COVID-19.

Persons who complete the questionnaire will be requested to give a blood sample to extract serum for the assessment of SARS-CoV-2 antibodies. Venous blood samples will be collected at the four different timepoints (Days 0, 21, 42, 63) to enable analysis in the laboratory. Blood will be collected by venous puncture performed by health workers within the participant’s home. In total, 4 ml of blood will be drawn in a 5 ml EDTA tube for each participant, at each timepoint.

The blood samples will be transported to the laboratory within 6 h of the collection, along with the relevant follow-up sheet for the blood samples. For transport to the laboratory, blood samples will be triple packed in an isotherm container containing an icebox to preserve the cold chain.

All blood samples will be analyzed in the Laboratory of the MURAZ center in Bobo-Dioulasso. Laboratory procedures involving handling of blood samples will be performed in a class 2 biosafety cabinet. For blood samples collected in the city of Bobo-Dioulasso, samples will be sent to the laboratory daily. Samples from Ouagadougou will also be sent daily to the laboratory of the national center for research and training on malaria in the same city where they will treated.

At the laboratory, plasma samples will be centrifuged and aliquoted within 12 h of the blood collection. If the time to preparation (centrifugation and aliquoting) is likely to exceed 12 h, it will be temporarily stored at + 4 °C for up to a maximum of 72 h. To minimize the number of freeze–thaw cycles [7], two (2) aliquots of 1 ml plasma each will be prepared from each blood sample. One of these will be used for the serological tests by ELISA anti-SARS-CoV-2 for both IgM and IgG. For participants who consent to conservation of their blood samples, the second aliquot will be stored at −80 °C in a secure biobank at the laboratory of the MURAZ center in Bobo-Dioulasso, in case needed for quality control, additional tests or future research.

Laboratory tests will use the WANTAI SARS-CoV-2 Ab ELISA, an Enzyme-Linked Immunosorbent Assay (ELISA) intended for qualitative detection of total antibodies (including IgG and IgM) to SARS-CoV-2 in human serum and Acid Citrate Dextrose (ACD) plasma. The WANTAI SARS-CoV-2 Ab ELISA is intended for use as an aid in identifying individuals with an adaptive immune response to SARS-CoV-2, indicating recent or prior infection. Before being used in this study, the assays will be validated for the Burkinabe context on a small series of characterized samples from Burkinabe patients (10 positive and 10 negative samples by PCR).

The results of the biological analyses and the questionnaire data will be recorded in an electronic case report form (CRF), which will be secured by personal access codes for each researcher. Data will be transferred to the data management center at the MURAZ center daily, after preliminary data checks by the supervisors in the field. Data will be cleaned and tested for coherence and completeness, and aberrant values will be identified. Queries generated in case of aberrant data will be sent to the research supervisors in the field to be resolved by the researchers before the end of data collection.

The database will be locked after the data has been cleaned and all queries have been resolved. The database will then be exported for statistical analysis.

### Statistical analysis

For the statistical analysis, this research project will adopt a dynamic approach and will constantly integrate latest knowledge emerging about COVID-19. A first analysis will be performed after the first collection on day 0, to enable important information that could be useful for the fight against COVID-19 to be relayed to the authorities in Burkina Faso.

The following analyses will be performed [7]:

- Calculation of the cumulative incidence by age group: i.e., the proportion of individuals who are seropositive for SARS-CoV-2 infection according to age (60 and over, 19–59 years old, teenagers aged 15–18 and children aged 10–14 years).
- Calculation of the cumulative incidence by gender: i.e., the proportion of individuals who are seropositive for SARS-CoV-2 infection according to gender (men, women).
- Serological response to infection (if the serological tests enable us to evaluate this criterion): i.e., the course of serum levels of specific antibodies against SARS-CoV-2 (increase in titers across the different measurement timepoints).
- Describe and compare individual, socio-economic characteristics, presence of comorbidities and exposure according to serological status of the population.

Quantitative variables will be described as mean ± standard deviation (SD) and median (range) and compared using the Student t or Mann–Whitney U tests as appropriate. Qualitative variables will be described as number and percentage and compared using the chi square or Fisher’s exact test, as appropriate.

## Phase 2: Clinical epidemiology

The design of this clinical epidemiological phase of the study will comprise an ambispective analysis, involving first, a retrospective cohort of all patients with COVID-19 followed in one of the dedicated COVID-19 centers in the cities of Ouagadougou or Bobo-Dioulasso since the beginning of the epidemic; and second, prospective inclusion of all patients who present up until the end of the epidemic, or at the latest, 6 months after the start of the study. Retrospective data will be collected after study initiation, from the medical files of patients diagnosed since the start of the epidemic but before the study was initiated. These data will be used (if possible, depending on the number of cases collected) to construct a score to predict the occurrence of severe complications and death related to COVID-19, which could then be used to optimize patient management. The score will be validated in the prospective cohort to be included after study initiation.

### Participant characteristics

The eligible population for this phase of the study will comprise all patients with a positive diagnosis of COVID-19 and followed in either of the two participating cities (Ouagadougou and Bobo-Dioulasso). In Burkina Faso, the epidemic is principally active in these two cities, the largest in the country, and the dedicated COVID-19 centers in these two cities manage almost all patients with a positive diagnosis since the start of the epidemic. The four main management sites are follows:

- In Ouagadougou: University hospital Yalgado, University hospital Bogodogo and University hospital Tengandogo;
- In Bobo Dioulasso: University hospital Sourou-Sanou.

### Inclusion criteria

- All patients followed for COVID-19 (whether they are or were hospitalized or not), in the reference management centers cited above, or in outpatient care, in Ouagadougou and Bobo-Dioulasso.

### Non-inclusion criteria

- Patients who refuse the use of their medical records for research purposes.

### Study endpoints

This phase of the study will have two primary endpoints, namely:

- The proportion of severe complications (defined as severe pneumonia, acute respiratory distress syndrome, sepsis or septic shock) in patients infected with SARS-CoV-2;
- The proportion of deaths in patients infected with SARS-CoV-2.

Secondary endpoints will be as follows:

- The proportion of severe complications according to age group and gender;
- The proportion of deaths according to age group and gender;

### Sample size calculation

No sample size can be calculated for this phase of the study. Exhaustive inclusion of all cases of COVID-19 followed in the cities of Ouagadougou and Bobo-Dioulasso is planned. At least 1,000 cases are anticipated (as of 28 November 2020, Burkina Faso had a total of 2,886 confirmed cases of COVID-19, of whom 984 were women and 1902 were men). The statistical power of the analyses will be calculated a posteriori*.*

### Data collection and management

Socio-demographic and clinical data (age, gender, comorbidities, vital signs, biological parameters) at the time of diagnosis of COVID-19 will be recorded by the clinical research assistants in the COVID-19 management centers from the patients’ medical files and recorded in the CRF for the study (paper format) (Additional file 2). The development of severe complications (type of complication, date, outcome) will also be recorded in the CRF. In case of death, the date of death will also be recorded. For patients who recover, the end-date for survival analysis will be the date of discharge from the COVID-19 management center.

Data will be recorded in a secure, electronic database using the in-house Ennov software from the Georges-François Leclerc Cancer Centre (Dijon, France). Data will be monitored before locking the database. Data will be analyzed at the Georges-François Leclerc Cancer Centre.

### Statistical analysis

Socio-demographic and clinical characteristics (age, gender, comorbidities, vital signs, biological parameters) of all patients at the time of diagnosis will be described and compared. Quantitative data will be described as mean ± SD or median (range). Qualitative variables will be described as number (percentage). Predictors of the occurrence of severe complications will be identified by logistic regression. The cohort will then be divided into two parts: the first part will be used to generate a score to predict severe complications (development set). The second part of the cohort will be used to validate the predictive score (validation set). The discriminatory capacity of the score will be assessed by the area under the receiver operating characteristic (ROC) curve (AUC), and the sensitivity, specificity, positive and negative predictive values of the score will be calculated. Prognostic factors of survival will be identified for the whole study population using Cox’s proportional hazards regression model. The outcome of interest will be death. Surviving patients will be censored at the date of discharge from the COVID-19 center. Imputation techniques and sensitivity analyses will be performed to account for missing data.

All analyses for phases 1 and 2 will be performed using STATA 14 (Copyright 1985–2015 StataCorp LLC; College Station, TX, USA) and R (www.r-project.org).

## Phase 3: Socio-anthropological study

The third and final phase of the study aims to analyze the healthcare pathway and the experiences of patients receiving care in dedicated centers for COVID-19 management in Burkina Faso, as well as the perceptions, acceptability, and application of preventive strategies in the population. An empirical study will be conducted in the COVID-19 management centers and in the community.

### Participant characteristics

For phase 3, sampling will take account of the different characteristics of the study zones and the population. The study will be performed in the cities of Ouagadougou and Bobo-Dioulasso, and four types of respondents will be included, namely:

- Persons who have recovered from COVID-19 after having been hospitalized in a specialized COVID-19 center. Eligible persons will be identified with help of the healthcare services;
- Relatives and/or caregivers (spouse, children etc.) of persons who have recovered from COVID-19 after having been treated in a specialized COVID-19 center;
- Healthcare professionals directly involved in the management of patients admitted to specialized COVID-19 centers (nurses, laboratory technicians, physicians);
- All-comers from the general population without known COVID-19 infection (men, women, young or old) in the cities of Ouagadougou and Bobo-Dioulasso.

For the choice of the study population, certain diversification criteria will be respected, to maximize heterogeneity, independently of the statistical frequency (young/old, men/women, city center/suburbs, nurses/laboratory technicians/physicians, high vs low level of education, etc.). The balance of these criteria will be verified as the study advances, during the daily and weekly updates. Similarly, the choice of the areas where the study will be carried out will aim to maximize the diversity of the different areas within both cities. The four following types of city area will be included: residential (central and peripheral), and poorer neighborhoods (central and peripheral). With four zones for each city, a total of eight zones will be included, as per the Table 1.

The choice of households will be at the discretion of the researcher in the field, according to the availability of persons to participate voluntarily in the study. The only restriction will be that the researchers must avoid including households who are already participating in the sero-epidemiological study, to avoid over-burdening the household members with research participation, and to prevent any influence of each study phase on the other.

### Inclusion criteria

Persons aged 18 years or older living in the cities of Ouagadougou or Bobo-Dioulasso:

- Who had received care for COVID-19 in one of the reference management centers, and who had recovered;
- Or a relative or caregiver (spouse, children etc.) Of such a person;
- Or a healthcare professional directly involved in the management of patients with COVID-19 in one of the reference management centers (nurses, laboratory technicians, physicians).

### Non-inclusion criteria

Persons who refuse to provide informed consent for study participation.

### Study endpoints

The endpoint for this study will be the acceptability and application of preventive strategies in the population.

### Sample size

For the sample size for phase 3, the principle of data saturation will be retained, i.e. the researchers will cease recruitment when it becomes clear that additional interviews and observations yield no new information either at individual or at group level. However, for the purposes of estimation, we aim to recruit around 100 persons altogether from the two cities. Persons who have recovered from COVID-19 infection will be identified via medical records by the medical staff, who will contact them to provide information about the study. The researcher will then contact the recovered patients thus identified, and invite them to participate in the study, and obtain the contact details of any relative or caregiver who could also be invited to participate. Healthcare professionals will be contacted directly in the COVID-19 management centers. Information about the study will be provided and their consent will be obtained. For the general population, researchers will recruit volunteers directly in their homes.

### Data collection and management

For this phase of the study, all persons who accept to participate (recovered patients, relatives/caregivers, medical staff) will be invited to participate in a semi-structured interview (Additional file 3) to explore their experience of COVID-19, treatment, the disease itself, their professional activity. Interviews will also explore perceptions, acceptability, and application of COVID-19 prevention strategies in the population.

### Analysis

Interviews will be recorded and complemented by observations made by the interviewer during the interview. Data will be transcribed and analyzed with the help of QDA Miner software.

## Discussion

### Communication and mobilization of the community

Given the nature of this study, and before the start of the sero-epidemiological (phase 1) and socio-anthropological (phase 3) components, we will openly dialogue with community leaders in the study zones. Through information to these leaders, to raise awareness about the positive repercussions of the study, we hope to ensure they will advocate for the study and convince the members of their communities to participate. Radio and/or television ads as well as public announcers with megaphones will also provide information to the public in the days prior to the researchers’ presence to collect data in the study zones. This widespread mobilization of the local community is key to achieving the inclusion and adherence necessary to guarantee the success of the study. In the general population, given that the blood draws will be performed in the participants’ homes, we plan to provide a small snack after the blood draw, comprised of a cold drink made of local fruit juices, and dry biscuits; these will be easy to handle without increasing the risk of contamination with SARS-CoV-2.

Participants in phase 2 (clinical epidemiology) who are hospitalized at the time of the study will receive written and oral information (patient information leaflet) about the study from a study investigator (physician). The information leaflet will outline the main objectives and procedures of the study and the expected risks and benefits. For participants who have difficulty understanding French, the written information will be complemented by oral information provided in the local language in the presence of a surrogate designated by the patient. For deceased patients, a request for exemption from the obligation to provide information will be submitted to the national ethics committee of Burkina Faso. The healthcare staff will try to contact as many recovered patients as possible to recover any data not available in the medical files, to ensure completeness of the CRFs, and minimize missing data. Recovered patients who accept to participate may come to the COVID-19 management center if they so desire to provide any data that is not available in the medical files. Their travel costs will be reimbursed in such cases.

### Protection of study researchers

The researchers collecting blood samples for the purposes of this study and those performing analyses in the laboratories will avail of WHO-recommended personal protective equipment including disposable gowns, medical masks, gloves, and visors. They will have permanent access to hydro-alcoholic gel to disinfect their hands. The researchers for phases 2 and 3 will also wear protective masks and will maintain social distancing (at least 1 m between persons).

All the persons included in the sero-epidemiological phase of the study, and any staff involved in the research who are found to be positive for SARS-CoV-2 during the study, despite protective hygiene measures, will be referred to the management center for appropriate care. The research team will ensure complete follow-up of anyone infected during the study activities.

### Implications

The results of this project will serve to shape public policy aimed at fighting the Covid-19 pandemic in Burkina Faso. The lessons learned from the management of the epidemic may serve as examples to the Burkinabe authorities to better design preventive strategies in the case of future epidemics or pandemics. To enable the dissemination and rapid use of the results of this study, we have already constituted a steering committee bringing together representatives from the Ministry for Health of Burkina Faso, experts in epidemiology, biology, politics, health systems and investigators for the current study. A non-medical organization that advocates for wider access to screening and treatment of COVID-19 (Réseau d’accès aux médicaments essentiels génériques) is also represented on the steering committee. The steering committee will meet every three months. During these meetings, the study investigators will provide an update on the advancement of the study procedures, and any difficulties encountered. The members of the committee will formulate recommendations for the implementation of the study, monitor study procedures, and provide advice about the interpretation of the findings that may be of use to decision-makers. The implication of political decision-makers, notably the Ministry for Health, in this study is a guarantee that the results will be rapidly translated into policy considerations to guide the strategy for response to the COVID-19 epidemic in Burkina Faso.

This research protocol can easily be adapted in any low-income country to generate accurate epidemiological, sociological, and anthropological data about the COVID-19 epidemic and adapt public health measure to control the disease. The lessons learned from the management of the epidemic may serve as examples to the Burkinabe authorities to better design preventive strategies in the case of future epidemics or pandemics.

## Supplementary Information

**Additional file 1.** English translation of the questionnaires related to the Phase 1: Sero-epidemiological study.

**Additional file 2.** English translation of the questionnaires related to the Phase 2: Clinical Epidemiology.

**Additional file 3.** English translation of the qualitative interview guide (Phase 3): general population.

## Data Availability

The data and materials will be made available on request.

## Abbreviations

ACD: Acid Citrate Dextrose
ANRS: France REcherche Nord&Sud Sida-hiv Hépatites
AUC: Area Under Curve
CAB: Cabinet
CRF: Case report form
EDTA: Ethylenediamine tetraacetic acid
ELISA: Enzyme-linked immunosorbent assay
HCQ-AZ: Combination of hydroxychloroquine plus azithromycin
IgG: Immunoglobulins G
IgM: Immunoglobulins M
INSP: Institut National de Santé Publique
LMICs: Low- and middle-income countries
mRNA: Messenger ribonucleic acid
MS: Ministry of Health
PCR: Polymerase Chain Reaction
ROC: Receiver operating characteristic
SARS-CoV-2: Severe acute respiratory syndrome coronavirus 2
TX: Texas
USA: United States of America
WHO: World Health Organization
