# Supplementary material for: COVID-19 epidemiological, sociological and anthropological investigation: study protocol for a multidisciplinary mixed methods research in Burkina Faso
Source: BMC Infect Dis. 2021 Sep 3;21:896. doi: 10.1186/s12879-021-06543-4 (PMC8414025; doi:10.1186/s12879-021-06543-4)
Supplement: Supplementary file 1 — Additional file 1. English translation of the questionnaires related to the Phase 1: Sero-epidemiological study. [file 12879_2021_6543_MOESM1_ESM.docx]

**Additional file 1: English translation of the questionnaires related to the Phase 1: Sero-epidemiological study**

**Multidisciplinary Study of COVID-19 in Burkina Faso**

**(EMuL-COVID-19), ANRS-COV13: sero- epidemiological survey**

**Inclusion Visit (D0)**

ID Participant: City: |____| Household: |____|____|____| Participant: |____|____|____|

| 1. **General Information** |
| --- |

| Date | └─┴─┘ └─┴─┘ └─┴─┴─┴─┘  Day/Month/Year | I1 |
| --- | --- | --- |
| Date of signature of informed consent | └─┴─┘ └─┴─┘ └─┴─┴─┴─┘  Day/Month/Year | I2 |
| Field investigator name (last and first name) |  | I3 |

| Study site | Ouagadougou 1  Bobo-Dioulasso 2 | I4 |
| --- | --- | --- |
| Participant household identification number |  | I5 |
| Participant identification number |  | I6 |
| Participant phone number |  | I7 |

| 1. **Socio-demographic Characteristics** | | |
| --- | --- | --- |
| **Question** | **Response** | **Code** |
| Sex? | Male 1 Female 2 | Q001 |
| Weight? | └─┴─┴─┘. └─┘ Kg | Q002 |
| Height? | └─┴─┴─┘cm | Q003 |
| How old are you?? | Years └─┴─┘ | Q004 |

ID Participant: City: |____| Household: |____|____|____| Participant: |____|____|____|

| Please indicate your marital status | Single 1  Married 2  Separated/divorced 3  Widowed 4  Living maritally 5  No response 77 | Q006 |
| --- | --- | --- |
| Which of the following categories best describes your main professional activity over the last 12 months? | Civil servant (State) 1  Civil servant (Private) 2  Tradesman 3  Volunteer 4  Student 5  Homemaker/housewife 6  Informal other than trade 7  Retired 8  Jobseeker 9  Invalidity 10  Other 11  If other, specify: …………………  No response 77 | Q007 |

ID Participant: City: |____| Household: |____|____|____| Participant: |____|____|____|

| 1. **Context and medical history (chronic disease/immunosuppression)** | | |
| --- | --- | --- |
| **Do you have any of the following diseases?** | | |
| **Question** | **Response** | **Code** |
| Diabetes? | Yes 1  No 2  Don’t know/ no answer 77  If yes, specify the type (1 or 2) if known: ………… | Q008 |
| Arterial hypertension? | Yes 1  No 2  Don’t know/ no answer 77 | Q009 |
| Chronic renal disease? | Yes 1  No 2  Don’t know/ no answer 77  If yes, specify: ……………………………………………. | Q010 |
| Chronic liver disease? | Yes 1  No 2  Don’t know/ no answer 77  If yes, specify: ……………………………………………. | Q011 |
| Chronic heart disease? | Yes 1  No 2  Don’t know/ no answer 77  If yes, specify: ……………………………………………. | Q012 |
| Chronic neurological or neuromuscular disease? | Yes 1  No 2  Don’t know/ no answer 77  If yes, specify: ……………………………………………. | Q012a |
| Chronic rheumatological disease? | Yes 1  No 2  Don’t know/ no answer 77  If yes, specify: ……………………………………………. | Q012b |
| HIV infection? | Yes 1  No 2  Don’t know/ no answer 77 | Q013 |
| Pulmonary tuberculosis? | Yes 1  No 2  Don’t know/ no answer 77 | Q014 |
| Other chronic respiratory diseases (asthma or COPD*)? | Yes 1  No 2  Don’t know/ no answer 77  if yes specify : | Q015 |

***COPD**: chronic obstructive pulmonary disease

ID Participant: City: |____| Household: |____|____|____| Participant: |____|____|____|

| Pregnancy? | Yes 1  No 2  Don’t know/ no answer / Not applicable 77  If yes, gestational age (weeks of amenorrhea) : | | Q016 |
| --- | --- | --- | --- |
| History of BCG vaccination documented/self-reported? | Yes (vaccination record) 1  Yes (injection site scar) 2  Yes (self-reported) 3  No 4 | | Q017 |
| Malignant tumor? | Yes 1  No 2  Don’t know/ no answer 77  if yes specify: | | Q018 |
| Other chronic disease (specify)?  …………………………………………… | Yes 1  No 2  Don’t know/ no answer 77 | | Q019 |
| Have you ever received a vaccine against COVID-19? | Yes (vaccination record) 1  Yes (injection site scar) 2  Yes (self-reported) 3  Specify if other document than the vaccination record  No (if no, go to question Q020) 4  Other 5  If other, specify: …………………………………… | Q019a | |
| If yes, |  | | |
| Specify if other document than the vaccination record |  | Q019b | |
| Specify the country |  | Q019c | |
| Number of doses received? | 1 or 2 | | Q019d |
| Origin of the vaccine received?  *Many possible answers* | Self-reported or reported by a relative 1  Vaccination card 2  Immunization Registry 3  Medical file 4  Other 5  If other, specify: ……………………………….. | | Q019e |
| Date of the 1^st^ dose?  Name of the vaccine: | └─┴─┘ └─┴─┘ └─┴─┴─┴─┘  Day/Month/Year  ………………………………………………………………………………. | Q019f  Q019g | |
| Date of the 2^nd^ dose?  Name of the vaccine : | └─┴─┘ └─┴─┘ └─┴─┴─┴─┘  Day/Month/Year  ………………………………………………………………………………. | Q019h  Q019i | |

ID Participant: City: |____| Household: |____|____|____| Participant: |____|____|____|

| 1. **Lifestyle and ongoing treatment** | | |
| --- | --- | --- |
| **Question** | **Response** | **Code** |
| Current smoker? | Yes 1  No 2  Don’t know/ no answer NK | Q020 |
| Former smoker? | Yes 1  No 2  Don’t know/ no answer NK | Q021 |
| If current or former smoker, duration of smoking? | └─┴─┘. └─┘ years | Q022 |
| How many cigarettes do (did) you smoker per day? | └─┴─┴─┘ cigarettes per day | Q023 |
| Within the past 12 months, how often did you have a drink containing alcohol? | Never 0  Once a month or less 1  2 to 4 times per month 2  2 to 3 times per week 3  At least 4 times per week 4  Don’t know/ no answer NK | Q024  ***If 0, go to Q028*** |
| How many drinks containing alcohol did you have on a typical day when you were drinking? | 1 or 2 0  3 or 4 1  5 or 6 2  7 to 9 3  10 or more 4  Don’t know/ no answer NK | Q025 |
| How often did you have six or more drinks on one occasion | Never 0  Once a month or less 1  2 to 4 times per month 2  2 to 3 times per week 3  At least 4 times per week 4  Don’t know/ no answer NK | Q026 |
| AUDIT Score, short version (sum of the scores for the 3 previous items) | └─┴─┘ | Q027 |
| Long term corticosteroid therapy (> 10 days of treatment)? | Yes 1  No 2  Don’t know/ no answer 77 | Q028 |
| Immunosuppressive therapy (radiotherapy or chemotherapy)? | Yes 1  No 2  Don’t know/ no answer 77 | Q029 |
| Ongoing antiretroviral therapy? | Yes 1  No 2  Don’t know/ no answer 77 | Q030 |
| Patient on dialysis? | Yes 1  No 2  Don’t know/ no answer 77 | Q031 |

ID Participant: City: |____| Household: |____|____|____| Participant: |____|____|____|

| 1. **Characterization of the exposure to COVID-19** | | |
| --- | --- | --- |
| **Question** | **Response** | **Code** |
| Have you ever had a nasopharyngeal swab for COVID-19? | Yes 1  No 2 If not go to the question Q036  Don’t know/ no answer 77 | Q032 |
| if yes, | | |
| Date? | └─┴─┘ └─┴─┘ └─┴─┴─┴─┘  Day/Month/Year | Q033 |
| Why? | Suspect case 1  Contact case 2  Border / airport control 3  Voluntary testing 4  Don’t know / no answer 77 | Q034 |
| What was the result? | Positive 1  Negative 2  Don’t know/ no answer 77 | Q035 |
| Self-medication (preventive or curative) against COVID-19? | Yes 1  No 2  Don’t know/ no answer 77 | Q036 |
| If yes, | | |
| Indicate the treatment taken | Chloroquine/hydroxychloroquine 1  Chloroquine/hydroxychloroquine + AZ* 2  Traditional herbal medicine 3  Other treatment (specify) 4  ………………………………………………………… | Q037  ***if 1,2 or 4, go to Q038*** |

***AZ**: Azithromycin

ID Participant: City: |____| Household: |____|____|____| Participant: |____|____|____|

| **USERS OF MEDICINAL PLANTS** | | |
| --- | --- | --- |
| **Question** | **Response** | **Code** |
| Was the herbal medicine prescribed by | Traditional practitioner 1  Grandma's Recipe 2  Other 3  If other, specify……………………………………….. | Q037a |

| Which plant (s) do you use or did you use for the treatment of covid-19? | Name of the plants | | **Parts of the plant used** | Q037b |
| --- | --- | --- | --- | --- |
|  | In local languages | Correspondence in French |  |  |
|  |  |  |  |  |
|  |  |  |  |  |
|  |  |  |  |  |
|  |  |  |  |  |
|  |  |  |  |  |

| Can you describe the preparation of the herbal medicine? (Carefully note all the steps and their duration) | ……………………………………………………………….  ………………………………………………………………  ………………………………………………………………  ………………………………………………………………  ………………………………………………………………  ……………………………………………………………….  ……………………………………………………………….  ……………………………………………………………….  ……………………………………………………………….  ……………………………………………………………….  ………………………………………………………………. | Q037c |
| --- | --- | --- |

ID Participant: City: |____| Household: |____|____|____| Participant: |____|____|____|

| What is the form of the phytomedicine at the time of its use? | Infusion 1  Decoction 2  Maceration 3  Smoke 4  Steam 5  Other 6  If other, specify……………………………………….. | Q037d |
| --- | --- | --- |
| How is the phytomedicine used? | Cold drink 1  Hot drink 2  Body bath 3  Inhalation / Fumigation 4  Application / Massage 5  Enema (purge) 6  Other mode of use 7  If other, specify ……………………………………….. |  |
| How long does the treatment last? | 1 to 3 days 1  4 to 7 days 2  1 week to 1 month 3  1 to 3 months 4  Several years 5 | Q037f |
| What is the dosage of the treatment?  Where do you find the treatment?  How did you use it? | ……………………………………………………………….  ………………………………………………………………  ……………………………………………………………  ……………………………………………………………….  ………………………………………………………………  ……………………………………………………………  ……………………………………………………………….  ………………………………………………………………  …………………………………………………………… | Q037g |
| Are there any prohibitions during the treatment? | Yes 1  No 2  Don’t know/ no answer 77 | Q037h |
| What are the prohibitions during the treatment? | ……………………………………………………………….  ………………………………………………………………  ………………………………………………………………  ……………………………………………………………… | Q037i |

ID Participant: City: |____| Household: |____|____|____| Participant: |____|____|____|

| **Did you in the last 14 days:** | | |
| --- | --- | --- |
| **Question** | **Response** | **Code** |
| Visit COVID-19 patients? | Yes 1  No 2  Don’t know/ no answer 77 | Q038 |
| Work with people with COVID-19? | Yes 1  No 2  Don’t know/ no answer 77 | Q039 |
| Have a face-to-face contact with a COVID-19 patient at a distance of less than 1m? | Yes 1  No 2  Don’t know/ no answer 77 | Q040 |
| Shared the same enclosed environment as a COVID-19 patient (including sharing a classroom or housekeeping or being at the same gathering)? | Yes 1  No 2  Don’t know/ no answer 77 | Q041 |
| Traveled (coach / bus / taxi / personal car / plane) with a COVID-19 patient? | Yes 1  No 2  Don’t know/ no answer 77 | Q042 |
| Provided direct care to COVID-19 patients? | Yes 1  No 2  Don’t know/ no answer 77 | Q043 |

ID Participant: City: |____| Household : |____|____|____| Participant : |____|____|____|

| 1. **Clinical Characteristics** | | | |
| --- | --- | --- | --- |
| **1st confirmed case of COVID-19 in Burkina Faso: 03/09/2020**  **During the past 14 days have you experienced / exhibited any of the following clinical signs:** | | | |
| **Question** | **Response** | **Code** | |
| Fever (≥38 ° C) or history of fever? | Yes 1  No 2  Don’t know/ no answer 77 | Q044 | |
| Asthenia / fatigue / malaise? | Yes 1  No 2  Don’t know/ no answer 77 | Q045 | |
| Myalgia / stiffness? | Yes 1  No 2  Don’t know/ no answer 77 | Q046 | |
| Joint pain? | Yes 1  No 2  Don’t know/ no answer 77 | Q047 | |
| Headache? | Yes 1  No 2  Don’t know/ no answer 77 | Q048 | |
| Chills (feeling cold)? | Yes 1  No 2  Don’t know/ no answer 77 | | Q049 |
| Cough? | Yes 1  No 2  Don’t know/ no answer 77 | | Q050 |
| Dyspnea (difficulty in breathing)? | Yes 1  No 2  Don’t know/ no answer 77 | | Q051 |
| Wheezing | Yes 1  No 2  Don’t know/ no answer 77 | | Q052 |
| Cold? | Yes 1  No 2  Don’t know/ no answer 77 | | Q053 |
| Epistaxis (nosebleed)? | Yes 1  No 2  Don’t know/ no answer 77 | | Q054 |
| Sore throat / angina? | Yes 1  No 2  Don’t know/ no answer 77 | | Q055 |
| Nausea / Vomiting? | Yes 1  No 2  Don’t know/ no answer 77 | | Q056 |
| Abdominal pain? | Yes 1  No 2  Don’t know/ no answer 77 | | Q057 |
| Diarrhea? | Yes 1  No 2  Don’t know/ no answer 77 | | Q058 |
| Conjunctivitis? | Yes 1  No 2  Don’t know/ no answer 77 | | Q059 |
| Ageusia (loss of taste)? | Yes 1  No 2  Don’t know/ no answer 77 | | Q060 |
| Anosmia (loss of smell)? | Yes 1  No 2  Don’t know/ no answer 77 | | Q061 |
| Anorexia (loss of appetite)? | Yes 1  No 2  Don’t know/ no answer 77 | | Q062 |
| Rash (rashes)? | Yes 1  No 2  Don’t know/ no answer 77 | | Q063 |
| Disorders of consciousness? | Yes 1  No 2  Don’t know/ no answer 77 | | Q064 |
| Convulsions? | Yes 1  No 2  Don’t know/ no answer 77 | | Q065 |
| Other symptoms (to be specified)?  …………………………………………… | Yes 1  No 2  Don’t know/ no answer 77 | | Q066 |

| **Complications** | | |
| --- | --- | --- |
| **Question** | **Response** | **Code** |
| Did you consult because of these symptoms / signs? | Yes 1  No 2  Don’t know/ no answer 77 | Q067 |
| Have these symptoms / signs caused you to miss school or work? | Yes 1  No 2  Don’t know/ no answer 77 | Q068 |
| Did these symptoms / signs require hospitalization? | Yes 1  No 2  Don’t know/ no answer 77 | Q069 |

ID Participant: City: |____| Household: |____|____|____| Participant: |____|____|____|

| 1. **Venous blood collection** | | |
| --- | --- | --- |
| **Question** | **Response** | **Code** |
| Blood sample taken? | Yes 1  No 2  If no, Why? ………………………………………..  ……………………………………………………………… | Q070 |
| Time of sampling? | └─┴─┘hours └─┴─┘minutes | Q070a |
| DBS realized? | Yes 1  No 2 | Q071 |
| Incident occurred during the sampling? | Yes 1  No 2  If yes, give details: ………………………………………..  ……………………………………………………………… | Q072 |

Name and surname of the field investigator Date and signature

ID Participant: City: |____| Household: |____|____|____| Participant: |____|____|____|

| 1. **D0 laboratory results** | | |
| --- | --- | --- |
| **Methods and results of serological tests** | | |
| **Question** | **Response** | **Code** |
| Sample number? | └─┴─┴─┴─┴─┘ | Q073 |
| Sampling date? | └─┴─┘ └─┴─┘ └─┴─┴─┴─┘  Day/Month/Year | Q074 |
| Time of sampling? | └─┴─┘hours └─┴─┘minutes | Q075 |
| Date received at the laboratory? | └─┴─┘ └─┴─┘ └─┴─┴─┴─┘  Day/Month/Year | Q076 |
| Reception time at the laboratory? | └─┴─┘hours └─┴─┘minutes | Q077 |
| Type of sample? | Serum 1  Other type (specify) 2  ……………………………………………………………… | Q078 |
| Result of the serological test? | IgG Positive  Negative    Undetermined    IgM Positive    Negative    Undetermined | Q079 |
| Result of the viral load? |  | Q080 |
| Sample transferred to another laboratory for confirmation? | Yes 1  No 2 | Q081 |
| if Yes, | | |
| Name and address of the laboratory for confirmation |  | Q082 |
| When was the sample sent? | └─┴─┘ └─┴─┘ └─┴─┴─┴─┘  Day/Month/Year | Q083 |
| When was the result confirmed? | └─┴─┘ └─┴─┘ └─┴─┴─┴─┘  Day/Month/Year | Q084 |
| What is the serological test confirmation result? | IgG Positive  Negative    Undetermined    IgM Positive    Negative    Undetermined | Q085 |

Name and surname of the biologist Date and signature

**ID Participant : City: |____| Household: |____|____|____| Participant: |____|____|____|**

| Name and surname of the participant |  |
| --- | --- |

**Multidisciplinary Study of COVID-19 in Burkina Faso**

**(EMuL-COVID-19), ANRS-COV13: sero- epidemiological survey**

**Follow-up visit (D21)**

| 1. **Characterization of the exposure to COVID-19** | | |
| --- | --- | --- |
| **Question** | **Response** | **Code** |
| Have you ever had a nasopharyngeal swab for COVID-19? | Yes 1  No 2 If not, go to the question Q090  Don’t know/ no answer 77 | Q086 |
| If yes, | | |
| Date? | └─┴─┘ └─┴─┘ └─┴─┴─┴─┘  Day/Month/Year | Q087 |
| Why? | Suspect case 1  Contact case 2  Border / airport control 3  Don’t know/ no answer 77 | Q088 |
| What was the result? | Positive 1  Negative 2  Don’t know/ no answer 77 | Q089 |
| Self-medication (preventive or curative) against COVID-19? | Yes 1  No 2  Don’t know/ no answer 77 | Q090 |
| If yes, | | |
| Indicate the treatment taken | Chloroquine/hydroxychloroquine 1  Chloroquine/hydroxychloroquine + AZ* 2  Traditional herbal medicine 3  Other treatment (specify) 4  ……………………………………………………………… | Q091  ***if 1,2 or 4, go to Q092*** |

***AZ**: Azithromycin

ID Participant: City: |____| Household: |____|____|____| Participant: |____|____|____|

| **USERS OF MEDICINAL PLANTS** | | |
| --- | --- | --- |
| **Question** | **Response** | **Code** |
| **Was the herbal medicine prescribed by** | Traditional practitioner 1  Grandma’s Recipe 2  Other 3  If other, specify……………………………………….. | Q091a |

| Which plant (s) do you use or did you use for the treatment of covid-19? | **Name of the plants** | | **Parts of the plant used** | Q091b |
| --- | --- | --- | --- | --- |
|  | In local languages | Correspondence in French |  |  |
|  |  |  |  |  |
|  |  |  |  |  |
|  |  |  |  |  |
|  |  |  |  |  |
|  |  |  |  |  |

| Can you describe the preparation of the herbal medicine? (Carefully note all the steps and their duration) | ……………………………………………………………….  ………………………………………………………………  ………………………………………………………………  ………………………………………………………………  ………………………………………………………………  ……………………………………………………………….  ……………………………………………………………….  ……………………………………………………………….  ……………………………………………………………….  ……………………………………………………………….  ………………………………………………………………. | Q091c |
| --- | --- | --- |

ID Participant: City: |____| Household: |____|____|____| Participant: |____|____|____|

| What is the form of the phytomedicine at the time of its use? | Infusion 1  Decoction 2  Maceration 3  Smoke 4  Steam 5  Other 6  If other, specify……………………………………….. | Q091d |
| --- | --- | --- |
| How is the phytomedicine used? | Cold drink 1  Hot drink 2  Body bath 3  Inhalation / Fumigation 4  Application/Massage 5  Enema (purge) 6  Other mode of use 7  If other, specify……………………………………….. | Q091e |
| How long does the treatment last? | 1 to 3 days 1  4 to 7 days 2  1 week to 1 month 3  1 to 3 months 4  Several years 5 | Q091f |
| What is the dosage of the treatment?  How did you use it? | ……………………………………………………………….  ………………………………………………………………  ………………………………………………………………  ………………………………………………………………  ………………………………………………………………  ……………………………………………………………….  ……………………………………………………………….  ………………………………………………………………  ……………………………………………………………….  ………………………………………………………………. | Q091g |

ID Participant: City: |____| Household: |____|____|____| Participant: |____|____|____|

| Are there any prohibitions during the treatment? | Yes 1  No 2  Don’t know/ no answer 77 | Q091h |
| --- | --- | --- |
| What are the prohibitions during the treatment? | ……………………………………………………………….  ………………………………………………………………  ………………………………………………………………  ……………………………………………………………… | Q091i |

| **Did you in the last 21 days (Since the last visit):** | | |
| --- | --- | --- |
| **Question** | **Response** | **Code** |
| Visit COVID-19 patients? | Yes 1  No 2  Don’t know/ no answer 77 | Q092 |
| Work with people with COVID-19? | Yes 1  No 2  Don’t know/ no answer 77 | Q093 |
| Have a face-to-face contact with a COVID-19 patient at a distance of less than 1m? | Yes 1  No 2  Don’t know/ no answer 77 | Q094 |
| Shared the same enclosed environment as a COVID-19 patient (including sharing a classroom or housekeeping or being at the same gathering)? | Yes 1  No 2  Don’t know/ no answer 77 | Q095 |
| Traveled (coach / bus / taxi / personal car / plane) with a COVID-19 patient? | Yes 1  No 2  Don’t know/ no answer 77 | Q096 |
| Provided direct care to COVID-19 patients? | Yes 1  No 2  Don’t know/ no answer 77 | Q097 |

ID Participant: City: |____| Household: |____|____|____| Participant: |____|____|____|

| 1. **Clinical Characteristics** | | |
| --- | --- | --- |
| **1st confirmed case of COVID-19 in Burkina Faso: 03/09/2020**  **During the past 21 days have you experienced / exhibited any of the following clinical signs** | | |
| **Question** | **Response** | **Code** |
| Fever (≥38 ° C) or history of fever? | Yes 1  No 2  Don’t know/ no answer 77 | Q098 |
| Asthenia / fatigue / malaise? | Yes 1  No 2  Don’t know/ no answer 77 | Q099 |
| Myalgia / stiffness? | Yes 1  No 2  Don’t know/ no answer 77 | Q100 |
| Joint pain? | Yes 1  No 2  Don’t know/ no answer 77 | Q101 |
| Headache? | Yes 1  No 2  Don’t know/ no answer 77 | Q102 |
| Chills (feeling cold)? | Yes 1  No 2  Don’t know/ no answer 77 | Q103 |
| Cough? | Yes 1  No 2  Don’t know/ no answer 77 | Q104 |
| Dyspnea (difficulty in breathing)? | Yes 1  No 2  Don’t know/ no answer 77 | Q105 |
| Wheezing | Yes 1  No 2  Don’t know/ no answer 77 | Q106 |
| Cold? | Yes 1  No 2  Don’t know/ no answer 77 | Q107 |
| Epistaxis (nosebleed)? | Yes 1  No 2  Don’t know/ no answer 77 | Q108 |
| Sore throat / angina? | Yes 1  No 2  Don’t know/ no answer 77 | Q109 |
| Nausea / Vomiting? | Yes 1  No 2  Don’t know/ no answer 77 | Q110 |

ID Participant: City: |____| Household: |____|____|____| Participant: |____|____|____|

| Abdominal pain? | Yes 1  No 2  Don’t know/ no answer 77 | Q111 |
| --- | --- | --- |
| Diarrhea? | Yes 1  No 2  Don’t know/ no answer 77 | Q112 |
| Conjunctivitis? | Yes 1  No 2  Don’t know/ no answer 77 | Q113 |
| Ageusia (loss of taste)? | Yes 1  No 2  Don’t know/ no answer 77 | Q114 |
| Anosmia (loss of smell)? | Yes 1  No 2  Don’t know/ no answer 77 | Q115 |
| Anorexia (loss of appetite)? | Yes 1  No 2  Don’t know/ no answer 77 | Q116 |
| Rash (rashes)? | Yes 1  No 2  Don’t know/ no answer 77 | Q117 |
| Disorders of consciousness? | Yes 1  No 2  Don’t know/ no answer 77 | Q118 |
| Convulsions? | Yes 1  No 2  Don’t know/ no answer 77 | Q119 |
| Other symptoms (to be specified)?  …………………………………………… | Yes 1  No 2  Don’t know/ no answer 77 | Q120 |
| Did you consult because of these symptoms / signs? | Yes 1  No 2  Don’t know/ no answer 77 | Q121 |
| Have these symptoms / signs caused you to miss school or work? | Yes 1  No 2  Don’t know/ no answer 77 | Q122 |
| Did these symptoms / signs require hospitalization? | Yes 1  No 2  Don’t know/ no answer 77 | Q123 |

ID Participant: City: |____| Household: |____|____|____| Participant: |____|____|____|

| 1. **Venous blood collection** | | |
| --- | --- | --- |
| **Question** | **Response** | **Code** |
| Blood sample taken? | Yes 1  No 2  If no, Why? ………………………………………..  ……………………………………………………………… | Q124 |
| Time of sampling? | └─┴─┘hours └─┴─┘minutes | Q124a |
| DBS realized? | Yes 1  No 2 | Q125 |
| Incident occurred during the sampling? | Yes 1  No 2  If yes, give details: ………………………………………..  ……………………………………………………………… | Q126 |

Name and surname of the field investigator Date and signature

ID Participant: City: |____| Household: |____|____|____| Participant: |____|____|____|

| 1. **laboratory results J21** | | |
| --- | --- | --- |
| **Méthodes and résultats des tests sérologiques** | | |
| **Question** | **Response** | **Code** |
| Sample number? | └─┴─┴─┴─┴─┘ | Q127 |
| Sampling date? | └─┴─┘ └─┴─┘ └─┴─┴─┴─┘  Day/Month/Year | Q128 |
| Time of sampling? | └─┴─┘hours └─┴─┘minutes | Q129 |
| Date received at the laboratory? | └─┴─┘ └─┴─┘ └─┴─┴─┴─┘  Day/Month/Year | Q130 |
| Reception time at the laboratory? | └─┴─┘hours └─┴─┘minutes | Q131 |
| Type of sample? | Serum 1  Other type (specify) 2  ……………………………………………………………… | Q132 |
| Result of the serological test? | IgG Positive  Negative    Undetermined    IgM Positive    Negative    Undetermined | Q133 |
| Result of the viral load? |  | Q134 |
| Sample transferred to another laboratory for confirmation? | Yes 1  No 2 | Q135 |
| If yes, | | |
| Name and address of the laboratory for confirmation |  | Q136 |
| When was the sample sent? | └─┴─┘ └─┴─┘ └─┴─┴─┴─┘  Day/Month/Year | Q137 |
| When was the result confirmed? | └─┴─┘ └─┴─┘ └─┴─┴─┴─┘  Day/Month/Year | Q138 |
| What is the serological test confirmation result? | IgG Positive  Negative    Undetermined    IgM Positive    Negative    Undetermined | Q139 |

Name and surname of the biologist Date and signature

**ID Participant: City: |____| Household: |____|____|____| Participant: |____|____|____|**

| Name and surname of the participant |  |
| --- | --- |

**Multidisciplinary Study of COVID-19 in Burkina Faso**

**(EMuL-COVID-19), ANRS-COV13: sero- epidemiological survey**

**Follow-up visit (D42)**

| 1. **Characterization of the exposure to COVID-19** | | |
| --- | --- | --- |
| **Question** | **Response** | **Code** |
| Have you ever had a nasopharyngeal swab for COVID-19? | Yes 1  No 2 If not, go to the question Q144  Don’t know/ no answer 77 | Q140 |
| If yes, | | |
| Date? | └─┴─┘ └─┴─┘ └─┴─┴─┴─┘  Day/Month/Year | Q141 |
| Why? | Suspect case 1  Contact case 2  Border / airport control 3  Don’t know/ no answer 77 | Q142 |
| What was the result? | Positive 1  Negative 2  Don’t know/ no answer 77 | Q143 |
| Self-medication (preventive or curative) against COVID-19? | Yes 1  No 2  Don’t know/ no answer 77 | Q144 |
| if yes, | | |
| Indicate the treatment taken | Chloroquine/hydroxychloroquine 1  Chloroquine/hydroxychloroquine + AZ* 2  Traditional herbal medicine 3  Other treatment (specify) 4 | Q145  ***if 1,2 or 4, go to Q146*** |

ID Participant: City: |____| Household : |____|____|____| Participant : |____|____|____|

| **USERS OF MEDICINAL PLANTS** | | |
| --- | --- | --- |
| **Question** | **Response** | **Code** |
| **Was the herbal medicine prescribed by** | Traditional practitioner 1  Grandma’s Recipe 2  Other 3  If other, specify……………………………………….. | Q145a |

| Which plant (s) do you use or did you use for the treatment of covid-19? | **Name of the plants** | | **Parts of the plant used** | Q145b |
| --- | --- | --- | --- | --- |
|  | In local languages | Correspondence in French |  |  |
|  |  |  |  |  |
|  |  |  |  |  |
|  |  |  |  |  |
|  |  |  |  |  |
|  |  |  |  |  |

| Can you describe the preparation of the herbal medicine? (carefully note all the steps and their duration) | ……………………………………………………………….  ………………………………………………………………  ………………………………………………………………  ………………………………………………………………  ………………………………………………………………  ……………………………………………………………….  ……………………………………………………………….  ……………………………………………………………….  ……………………………………………………………….  ………………………………………………………………. | Q145c |
| --- | --- | --- |

ID Participant: City: |____| Household: |____|____|____| Participant : |____|____|____|

| What is the form of the phytomedicine at the time of its use? | | Infusion 1  Decoction 2  Maceration 3  Smoke 4  Steam 5  Other 6  If other, specify……………………………………….. | Q145d |
| --- | --- | --- | --- |
| How is the phytomedicine used? | | Cold drink 1  Hot drink 2  Body bath 3  Inhalation / Fumigation 4  Application/Massage 5  Enema (purge) 6  Other mode of use 7  If other, specify……………………………………….. | Q145e |
| How long does the treatment last? | | 1 to 3 days 1  4 to 7 days 2  1 week to 1 month 3  1 to 3 months 4  Several years 5 | Q145f |
| What is the dosage of the treatment?  What is the dosage of the treatment?  How did you use it? | ……………………………………………………………….  ………………………………………………………………  ………………………………………………………………  ………………………………………………………………  ………………………………………………………………  ……………………………………………………………….  ……………………………………………………………….  ………………………………………………………………. | | Q145g |

ID Participant: City: |____| Household : |____|____|____| Participant : |____|____|____|

| Are there any prohibitions during the treatment? | Yes 1  No 2  Don’t know/ no answer 77 | Q145h |
| --- | --- | --- |
| What are the prohibitions during the treatment? | ……………………………………………………………….  ………………………………………………………………  ………………………………………………………………  ……………………………………………………………… | Q145i |

| **Did you in the last 21 days:** | | |
| --- | --- | --- |
| **Question** | **Response** | **Code** |
| Visit COVID-19 patients? | Yes 1  No 2  Don’t know/ no answer 77 | Q146 |
| Work with people with COVID-19? | Yes 1  No 2  Don’t know/ no answer 77 | Q147 |
| Have a face-to-face contact with a COVID-19 patient at a distance of less than 1m? | Yes 1  No 2  Don’t know/ no answer 77 | Q148 |
| Shared the same enclosed environment as a COVID-19 patient (including sharing a classroom or housekeeping or being at the same gathering)? | Yes 1  No 2  Don’t know/ no answer 77 | Q149 |
| Traveled (coach / bus / taxi / personal car / plane) with a COVID-19 patient? | Yes 1  No 2  Don’t know/ no answer 77 | Q150 |
| Provided direct care to COVID-19 patients? | Yes 1  No 2  Don’t know/ no answer 77 | Q151 |

ID Participant: City: |____| Household: |____|____|____| Participant : |____|____|____|

| 1. **Clinical Characteristics** | | | | |
| --- | --- | --- | --- | --- |
| **1st confirmed case of COVID-19 in Burkina Faso: 03/09/2020**  **During the past 21 days have you experienced / exhibited any of the following clinical sign** | | | | |
| **Question** | **Response** | | **Code** | |
| Fever (≥38 ° C) or history of fever? | Yes 1  No 2  Don’t know/ no answer 77 | | Q152 | |
| Asthenia / fatigue / malaise? | Yes 1  No 2  Don’t know/ no answer 77 | | Q153 | |
| Myalgia / stiffness? | Yes 1  No 2  Don’t know/ no answer 77 | Q154 | | |
| Joint pain? | Yes 1  No 2  Don’t know/ no answer 77 | Q155 | | |
| Headache? | Yes 1  No 2  Don’t know/ no answer 77 | | Q156 | |
| Chills (feeling cold)? | Yes 1  No 2  Don’t know/ no answer 77 | Q157 | | |
| Cough? | Yes 1  No 2  Don’t know/ no answer 77 | | Q158 | |
| Dyspnea (difficulty in breathing)? | Yes 1  No 2  Don’t know/ no answer 77 | Q159 | | |
| Wheezing | Yes 1  No 2  Don’t know/ no answer 77 | Q160 | | |
| Cold? | Yes 1  No 2  Don’t know/ no answer 77 | | Q161 | |
| Epistaxis (nosebleed)? | Yes 1  No 2  Don’t know/ no answer 77 | | | Q162 |
| Sore throat / angina? | Yes 1  No 2  Don’t know/ no answer 77 | | | Q163 |
| Nausea / Vomiting? | Yes 1  No 2  Don’t know/ no answer 77 | | | Q164 |

ID Participant: City: |____| Household: |____|____|____| Participant: |____|____|____|

| Abdominal pain? | Yes 1  No 2  Don’t know/ no answer 77 | Q165 |
| --- | --- | --- |
| Diarrhea? | Yes 1  No 2  Don’t know/ no answer 77 | Q166 |
| Conjunctivitis? | Yes 1  No 2  Don’t know/ no answer 77 | Q167 |
| Ageusia (loss of taste)? | Yes 1  No 2  Don’t know/ no answer 77 | Q168 |
| Anosmia (loss of smell)? | Yes 1  No 2  Don’t know/ no answer 77 | Q169 |
| Anorexia (loss of appetite)? | Yes 1  No 2  Don’t know/ no answer 77 | Q170 |
| Rash (rashes)? | Yes 1  No 2  Don’t know/ no answer 77 | Q171 |
| Disorders of consciousness? | Yes 1  No 2  Don’t know/ no answer 77 | Q172 |
| Convulsions? | Yes 1  No 2  Don’t know/ no answer 77 | Q173 |
| Other symptoms (to be specified)?  …………………………………………… | Yes 1  No 2  Don’t know/ no answer 77 | Q174 |
| Did you consult because of these symptoms / signs? | Yes 1  No 2  Don’t know/ no answer 77 | Q175 |
| Have these symptoms / signs caused you to miss school or work? | Yes 1  No 2  Don’t know/ no answer 77 | Q176 |
| Did these symptoms / signs require hospitalization? | Yes 1  No 2  Don’t know/ no answer 77 | Q177 |

| 1. **Venous blood collection** | | |
| --- | --- | --- |
| **Question** | **Response** | **Code** |
| Blood sample taken? | Yes 1  No 2  If no, Why? ………………………………………..  ……………………………………………………………… | Q178 |
| Time of sampling? | └─┴─┘hours └─┴─┘minutes | Q178a |
| DBS realized? | Yes 1  No 2 | Q179 |
| Incident occurred during the sampling? | Yes 1  No 2  If yes, give details: ………………………………………..  ……………………………………………………………… | Q180 |

Name and surname of the field investigator Date and signature

ID Participant: City: |____| Household: |____|____|____| Participant: |____|____|____|

| 1. **D42 laboratory results** | | |
| --- | --- | --- |
| **Methods and results of serological tests (a new table for each sample collected)** | | |
| **Question** | **Response** | **Code** |
| Sample number? |  | Q181 |
| Sampling date? | └─┴─┘ └─┴─┘ └─┴─┴─┴─┘  Day/Month/Year | Q182 |
| Time of sampling? | └─┴─┘hours └─┴─┘minutes | Q183 |
| Date received at the laboratory? | └─┴─┘ └─┴─┘ └─┴─┴─┴─┘  Day/Month/Year | Q184 |
| Reception time at the laboratory? | └─┴─┘hours └─┴─┘minutes | Q185 |
| Type of sample? | Serum 1  Other type (specify) 2  ……………………………………………………………… | Q186 |
| Result of the serological test? | IgG Positive  Negative    Undetermined    IgM Positive    Negative    Undetermined | Q187 |
| Result of the viral load? |  | Q188 |
| Sample transferred to another laboratory for confirmation? | Yes 1  No 2 | Q189 |
| if Yes, | | |
| Name and address of the laboratory for confirmation |  | Q190 |
| When was the sample sent? | └─┴─┘ └─┴─┘ └─┴─┴─┴─┘  Day/Month/Year | Q191 |
| When was the result confirmed? | └─┴─┘ └─┴─┘ └─┴─┴─┴─┘  Day/Month/Year | Q192 |
| What is the serological test confirmation result? | IgG Positive  Negative    Undetermined    IgM Positive    Negative    Undetermined | Q193 |

**ID Participant : City: |____| Household: |____|____|____| Participant: |____|____|____|**

| Name and surname of the participant |  |
| --- | --- |

**Multidisciplinary Study of COVID-19 in Burkina Faso**

**(EMuL-COVID-19), ANRS-COV13: sero- epidemiological survey**

**Follow-up visit (D63)**

| 1. **Characterization of the exposure to COVID-19** | | | |
| --- | --- | --- | --- |
| **Question** | **Response** | **Code** | |
| Have you ever had a nasopharyngeal swab for COVID-19? | Yes 1  No 2 If no, go to the question Q198  Don’t know/ no answer 77 | Q194 | |
| If yes, | | | |
| Date? | └─┴─┘ └─┴─┘ └─┴─┴─┴─┘  Day/Month/Year | Q195 | |
| Why? | Suspect case 1  Contact case 2  Border / airport control 3  Don’t know/ no answer 77 | Q196 | |
| What was the result? | Positive 1  Negative 2  Don’t know/ no answer 77 | Q197 | |
| Self-medication (preventive or curative) against COVID-19? | Yes 1  No 2  Don’t know/ no answer 77 | Q198 |  |
| If yes, | | |  |
| Indicate the treatment taken | Chloroquine/hydroxychloroquine 1  Chloroquine/hydroxychloroquine + AZ* 2  Traditional herbal medicine 3  Other treatment (specify) 4  ……………………………………………………………… | Q199  ***if 1,2 or 4, go to Q200*** |  |

***AZ**: Azithromycin

ID Participant: City: |____| Household: |____|____|____| Participant: |____|____|____|

| **USERS OF MEDICINAL PLANTS** | | |
| --- | --- | --- |
| **Question** | **Response** | **Code** |
| **Was the herbal medicine prescribed by** | Traditional practitioner 1  Grandma’s Recipe 2  Other 3  If other, specify……………………………………….. | Q199a |

| Which plant (s) do you use, or did you use for the treatment of covid-19? | **Name of the plants** | | **Parts of the plant used** | Q199b |
| --- | --- | --- | --- | --- |
|  | In local languages | Correspondence in French |  |  |
|  |  |  |  |  |
|  |  |  |  |  |
|  |  |  |  |  |
|  |  |  |  |  |
|  |  |  |  |  |

| Can you describe the preparation of the herbal medicine? (carefully note all the steps and their duration) | ……………………………………………………………….  ………………………………………………………………  ………………………………………………………………  ………………………………………………………………  ………………………………………………………………  ……………………………………………………………….  ……………………………………………………………….  ……………………………………………………………….  ……………………………………………………………….  ……………………………………………………………….  ………………………………………………………………. | Q199c |
| --- | --- | --- |

ID Participant: City: |____| Household: |____|____|____| Participant: |____|____|____|

| What is the form of the phytomedicine at the time of its use? | | Infusion 1  Decoction 2  Maceration 3  Smoke 4  Steam 5  Other 6  If other, specify……………………………………….. | Q199d |
| --- | --- | --- | --- |
| How is the phytomedicine used? | | Cold drink 1  Hot drink 2  Body bath 3  Inhalation / Fumigation 4  Application/Massage 5  Enema (purge) 6  Other mode of use 7  If other, specify……………………………………….. | Q199e |
| How long does the treatment last? | | 1 to 3 days 1  4 to 7 days 2  1 week to 1 month 3  1 to 3 months 4  Several years 5 | Q199f |
| What is the dosage of the treatment?  What is the dosage of the treatment?  How did you use it? | ……………………………………………………………….  ………………………………………………………………  ………………………………………………………………  ………………………………………………………………  ………………………………………………………………  ……………………………………………………………….  ……………………………………………………………….  ……………………………………………………………….  ……………………………………………………………….  ……………………………………………………………….  ………………………………………………………………. | | Q199g |

ID Participant: City: |____| Household: |____|____|____| Participant: |____|____|____|

| Are there any prohibitions during the treatment? | Yes 1  No 2  Don’t know/ no answer 77 | Q199h |
| --- | --- | --- |
| What are the prohibitions during the treatment? | ……………………………………………………………….  ………………………………………………………………  ………………………………………………………………  ……………………………………………………………… | Q199i |

| **Did you in the last 21 days:** | | |
| --- | --- | --- |
| **Question** | **Response** | **Code** |
| Visit COVID-19 patients? | Yes 1  No 2  Don’t know/ no answer 77 | Q200 |
| Work with people with COVID-19? | Yes 1  No 2  Don’t know/ no answer 77 | Q201 |
| Have a face-to-face contact with a COVID-19 patient at a distance of less than 1m? | Yes 1  No 2  Don’t know/ no answer 77 | Q202 |
| Shared the same enclosed environment as a COVID-19 patient (including sharing a classroom or housekeeping or being at the same gathering)? | Yes 1  No 2  Don’t know/ no answer 77 | Q203 |
| Traveled (coach / bus / taxi / personal car / plane) with a COVID-19 patient? | Yes 1  No 2  Don’t know/ no answer 77 | Q204 |
| Provided direct care to COVID-19 patients? | Yes 1  No 2  Don’t know/ no answer 77 | Q205 |

ID Participant: City: |____| Household: |____|____|____| Participant: |____|____|____|

| 1. **Clinical Characteristics** | | | | |
| --- | --- | --- | --- | --- |
| **1st confirmed case of COVID-19 in Burkina Faso: 03/09/2020**  **During the past 21 days have you experienced / exhibited any of the following clinical signs:** | | | | |
| **Question** | **Response** | | **Code** | |
| Fever (≥38 ° C) or history of fever? | Yes 1  No 2  Don’t know/ no answer 77 | | Q206 | |
| Asthenia / fatigue / malaise? | Yes 1  No 2  Don’t know/ no answer 77 | | Q207 | |
| Myalgia / stiffness? | Yes 1  No 2  Don’t know/ no answer 77 | Q208 | | |
| Joint pain? | Yes 1  No 2  Don’t know/ no answer 77 | Q209 | | |
| Headache? | Yes 1  No 2  Don’t know/ no answer 77 | | Q210 | |
| Chills (feeling cold)? | Yes 1  No 2  Don’t know/ no answer 77 | Q211 | | |
| Cough? | Yes 1  No 2  Don’t know/ no answer 77 | | Q212 | |
| Dyspnea (difficulty in breathing)? | Yes 1  No 2  Don’t know/ no answer 77 | Q213 | | |
| Wheezing | Yes 1  No 2  Don’t know/ no answer 77 | Q214 | | |
| Cold? | Yes 1  No 2  Don’t know/ no answer 77 | | Q215 | |
| Epistaxis (nosebleed)? | Yes 1  No 2  Don’t know/ no answer 77 | | | Q216 |
| Sore throat / angina? | Yes 1  No 2  Don’t know/ no answer 77 | | | Q217 |

ID Participant: City: |____| Household: |____|____|____| Participant: |____|____|____|

| Nausea / Vomiting? | Yes 1  No 2  Don’t know/ no answer 77 | Q218 |
| --- | --- | --- |
| Abdominal pain? | Yes 1  No 2  Don’t know/ no answer 77 | Q219 |
| Diarrhea? | Yes 1  No 2  Don’t know/ no answer 77 | Q220 |
| Conjunctivitis? | Yes 1  No 2  Don’t know/ no answer 77 | Q221 |
| Ageusia (loss of taste)? | Yes 1  No 2  Don’t know/ no answer 77 | Q222 |
| Anosmia (loss of smell)? | Yes 1  No 2  Don’t know/ no answer 77 | Q223 |
| Anorexia (loss of appetite)? | Yes 1  No 2  Don’t know/ no answer 77 | Q224 |
| Rash (rashes)? | Yes 1  No 2  Don’t know/ no answer 77 | Q225 |
| Disorders of consciousness? | Yes 1  No 2  Don’t know/ no answer 77 | Q226 |
| Convulsions? | Yes 1  No 2  Don’t know/ no answer 77 | Q227 |
| Other symptoms (to be specified)?  …………………………………………… | Yes 1  No 2  Don’t know/ no answer 77 | Q228 |
| Did you consult because of these symptoms / signs? | Yes 1  No 2  Don’t know/ no answer 77 | Q229 |
| Have these symptoms / signs caused you to miss school or work? | Yes 1  No 2  Don’t know/ no answer 77 | Q230 |
| Did these symptoms / signs require hospitalization? | Yes 1  No 2  Don’t know/ no answer 77 | Q231 |

ID Participant: City: |____| Household: |____|____|____| Participant: |____|____|____|

| 1. **Venous blood collection** | | |
| --- | --- | --- |
| **Question** | **Response** | **Code** |
| Blood sample taken? | Yes 1  No 2  If no, Why? ………………………………………..  ……………………………………………………………… | Q232 |
| Time of sampling? | └─┴─┘hours └─┴─┘minutes | Q232a |
| DBS realized? | Yes 1  No 2 | Q233 |
| Incident occurred during the sampling? | Yes 1  No 2  If yes, give details: ………………………………………..  ……………………………………………………………… | Q234 |

Name and surname of the field investigator Date and signature

ID Participant: City: |____| Household: |____|____|____| Participant: |____|____|____|

| 1. **D63 laboratory results** | | |
| --- | --- | --- |
| **Methods and results of serological tests (a new table for each sample collected)** | | |
| **Question** | **Response** | **Code** |
| Sample number? | └─┴─┴─┴─┴─┘ | Q235 |
| Sampling date? | └─┴─┘ └─┴─┘ └─┴─┴─┴─┘  Day/Month/Year | Q236 |
| Time of sampling? | └─┴─┘hours └─┴─┘minutes | Q237 |
| Date received at the laboratory? | └─┴─┘ └─┴─┘ └─┴─┴─┴─┘  Day/Month/Year | Q238 |
| Reception time at the laboratory? | └─┴─┘hours └─┴─┘minutes | Q239 |
| Type of sample? | Serum 1  Other type (specify) 2 | Q240 |
| Result of the serological test? | IgG Positive  Negative    Undetermined    IgM Positive    Negative    Undetermined | Q241 |
| Result of the viral load? |  | Q242 |
| Sample transferred to another laboratory for confirmation? | Yes 1  No 2 | Q243 |
| if Yes, | | |
| Name and address of the laboratory for confirmation |  | Q244 |
| When was the sample sent? | └─┴─┘ └─┴─┘ └─┴─┴─┴─┘  Day/Month/Year | Q245 |
| When was the result confirmed? | └─┴─┘ └─┴─┘ └─┴─┴─┴─┘  Day/Month/Year | Q246 |
| What is the serological test confirmation result? | IgG Positive  Negative    Undetermined    IgM Positive    Negative    Undetermined | Q247 |

Name and surname of the biologist Date and signature
