# Supplementary material for: COVID-19 epidemiological, sociological and anthropological investigation: study protocol for a multidisciplinary mixed methods research in Burkina Faso
Source: BMC Infect Dis. 2021 Sep 3;21:896. doi: 10.1186/s12879-021-06543-4 (PMC8414025; doi:10.1186/s12879-021-06543-4)
Supplement: Supplementary file 2 — Additional file 2. English translation of the questionnaires related to the Phase 2: Clinical Epidemiology. [file 12879_2021_6543_MOESM2_ESM.docx]

**Additional file 2: English translation of the questionnaires related to the Phase 2: Clinical Epidemiology**

**Multidisciplinary Study of COVID-19 in Burkina Faso**

**(EMuL-COVID-19), ANRS-COV13 : Clinical Epidemiology**

**Inclusion Visit (D0)**

| 1. **General Information** |
| --- |

| Date | └─┴─┘ └─┴─┘ └─┴─┴─┴─┘  Day/Month/Year | I1 |
| --- | --- | --- |
| Date of signature of informed consent (if applicable) | └─┴─┘ └─┴─┘ └─┴─┴─┴─┘  Day/Month/Year | I2 |

| Study site | Ouagadougou 1  Bobo-Dioulasso 2 | I3 |
| --- | --- | --- |
| District / area of residence |  | I4 |
| Health authority area of residence |  | I5 |
| Where is the participant followed-up? | Reference Centre 1 Home 2  Hotel/shelter 3  Clinic 4 | I6 |
| Name of place of follow-up (if not the participant’s home) |  | I7 |
| Date of admission / date of first visit | └─┴─┘ └─┴─┘ └─┴─┴─┴─┘  Day/Month/Year | I8 |
| Participant identification number | \|____\|____\|____\| | I9 |
| Patient inclusion number (To be noted on the confidential list of correspondence) | \|___\|___\| \|___\| \|__\| \|___\|___\|  └─┴─┴─┴─┘ | I9bis |
| Participant’s phone number and/or  Phone number of accompanying person |  | I10 |
| Documented SARS-CoV-2 reinfection? | Yes 1  No 2 | I11 |
| If yes, please indicate the name of the first centre where the patient was managed, and the number of the medical file |  | I12 |

| 1. **Socio-demographic Characteristics** | | |
| --- | --- | --- |
| **Question** | **Response** | **Code** |
| Sex ? | Male 1 Female 2 | Q001 |
| Place of residence ? | Urban 1 Rural 2 | Q002 |
| How old are you ? | Years ☐└─┴─┘  Months ☐ | Q003 |
| Please indicate the highest level of education you attained | No school 1  Basic literacy 2  Primary school 3  Junior high school 4  Senior high school 5  University 6  Other 7  If other, specify :………………  No response NK | Q004 |
| Please indicate your marital status | Single 1  Married 2  Separated/divorced 3  Widowed 4  Living maritally 5    No response NK | Q005 |
| Which of the following categories best describes your **main** **professional activity** over the last 12 months? | Civil servant (State) 1  Civil servant (Private) 2  Tradesman 3  Volunteer 4  Student 5  Homemaker/housewife 6  Informal other than trade 7  Retired 8  Jobseeker 9  Invalidity 10  Other 11  If other, specify :…………………  No response NK | Q006 |

ID Participant : Site : |____| Participant : |____|____|____|

| Laboratory professional? | Yes 1  No 2 | Q007 |
| --- | --- | --- |
| Healthcare professional? | Yes 1  No 2 | Q008 |
| Veterinary health professional? | Yes 1  No 2 | Q009 |

| 1. **Context and medical history (chronic disease/immunosuppression)** | | |
| --- | --- | --- |
| **Do you have any of the following diseases?** | | |
| **Question** | **Response** | **Code** |
| Diabetes mellitus? | Yes 1  No 2  Don’t know/ no answer NK  If yes, specify type I or type II, if known:………… | Q010 |
| Arterial hypertension? | Yes 1  No 2  Don’t know/ no answer NK | Q011 |
| Cardiovascular disease other than arterial hypertension? | Yes 1  No 2  Don’t know/ no answer NK  If yes, specify :……………………………………………. | Q012 |
| Chronic renal disease? | Yes 1  No 2  Don’t know/ no answer NK  If yes, specify :……………………………………………. | Q013 |
| Chronic liver disease? | Yes 1  No 2  Don’t know/ no answer NK  If yes, specify :……………………………………………. | Q014 |
| Chronic neurological or neuromuscular disease? | Yes 1  No 2  Don’t know/ no answer NK  If yes, specify :……………………………………………. | Q015 |
| Chronic rheumatological disease? | Yes 1  No 2  Don’t know/ no answer NK  If yes, specify :……………………………………………. | Q016 |

ID Participant: Site : |____| Participant : |____|____|____|

| HIV infection? | Yes 1  No 2  Don’t know/ no answer NK | Q017 |
| --- | --- | --- |
| Pulmonary tuberculosis? | Yes 1  No 2  Don’t know/ no answer NK | Q018 |
| Other chronic respiratory diseases (asthma or COPD*)? | Yes 1  No 2  Don’t know/ no answer NK  If yes, specify :……………………………………………. | Q019 |
| Pregnancy? | Yes 1  No 2  Don’t know/ no answer NK  Not applicable NA  If yes, gestational age (weeks of amenorrhea) :└─┴─┘ | Q020 |
| Post-partum (delivery < 42 days) ? | Yes 1  No 2  Don’t know/ no answer NK  Not applicable NA | Q021 |
| History of BCG vaccination documented/self-reported? | Yes (vaccination record) 1  Yes (injection site scar) 2  Yes (self-reported) 3  No 4 | Q022 |
| History of blood transfusion ? | Yes 1  No 2  Don’t know/ no answer NK  If yes, specify last known date:    └─┴─┘ └─┴─┘ └─┴─┴─┴─┘  Day/Month/Year | Q023 |
| Malignant tumor ? | Yes 1  No 2  Don’t know/ no answer NK  If yes, specify:……………………………………………. | Q024 |
| Other chronic disease (specify) ?  …………………………………………… | Yes 1  No 2  Don’t know/ no answer NK | Q025 |

***COPD** : chronic obstructive pulmonary disease

ID Participant : Site : |____| Participant : |____|____|____|

| 1. **Lifestyle and ongoing treatment** | | | |
| --- | --- | --- | --- |
| **Question** | **Response** | | **Code** |
| Current smoker ? | Yes 1  No 2  Don’t know/ no answer NK | | Q026 |
| Former smoker ? | Yes 1  No 2  Don’t know/ no answer NK | | Q027 |
| If current or former smoke, duration of smoking? | └─┴─┘, └─┘ years | | Q028 |
| How many cigarettes do (did) you smoke per day ? | └─┴─┴─┘ cigarettes per day | | Q029 |
| How often did you consume alcoholic drinks over the last 12 months ? | Never 0  Once a month or less 1  2 to 4 times per month 2  2 to 3 times per week 3  At least 4 times per week 4 | | Q030  **If 0,  go to Q033** |
| How many standard glasses do you drink on an average day where you consume alcohol ? | 1 or 2 0  3 or 4 1  5 or 6 2  7 to 9 3  10 or more 4 | Q031 | |
| How often would you drink six standard glasses or more, on a single occasion? | Never 0  Once a month or less 1  2 to 4 times per month 2  2 to 3 times per week 3  At least 4 times per week 4 | Q032 | |
| AUDIT Score, short version (sum of the scores for the 3 previous items) | └─┴─┘ | Q033 | |
| Long term corticoid therapy (> 10 days of treatment)? | Yes 1  No 2  Don’t know/ no answer NK | Q034 | |
| Immunosuppressive therapy (radiotherapy or chemotherapy) ? | Yes 1  No 2  Don’t know/ no answer NK | Q035 | |
| Ongoing antiretroviral therapy? | Yes 1  No 2  Don’t know/ no answer NK | Q036 | |
| Patient on dialysis? | Yes 1  No 2  Don’t know/ no answer NK | Q037 | |

ID Participant: Site : |____| Participant : |____|____|____|

| Self-medication (preventive or curative) against COVID-19? | Yes 1  No 2  No answer NK | Q038 |
| --- | --- | --- |
| If yes, | | |
| Indicate the treatment taken | Chloroquine/hydroxychloroquine 1  Chloroquine/hydroxychloroquine + AZ* 2  Traditional phytotherapy 3  Other (specify) 4  ……………………………………………………………… | Q039 |

***AZ** : Azithromycin

| 1. **Mode of admission and diagnosis** | | |
| --- | --- | --- |
| **Question** | **Response** | **Code** |
| Mode of admission? | Direct admission 1  Referred by another health establishment 2  Patient not hospitalized 3  Don’t know / no answer NK | Q040 |
| Test in a contact case ? | Yes 1  No 2  Don’t know/ no answer NK | Q041 |
| Test in a suspected case ? | Yes 1  No 2  Don’t know/ no answer NK | Q042 |
| Clinical signs without known contact with a confirmed case of COVID-19 ? | Yes 1  No 2  Don’t know/ no answer NK | Q043 |
| Screening | Yes 1  No 2  Don’t know/ no answer NK | Q044 |
| If yes, specify : | Voluntary screening 1  Travelling outbound 2  Screening required by employer 3  Systematic at border control (incoming traveler) 4 | Q045 |
| If incoming traveler, specify country of origin? |  | Q046 |

ID Participant : Site : |____| Participant : |____|____|____|

| Date of sample retrieval for PCR ? | └─┴─┘ └─┴─┘ └─┴─┴─┴─┘  Day/Month/Year | Q047 |
| --- | --- | --- |
| Type of sample ? | Nasopharyngeal 1  Oropharyngeal 2  Nasopharyngeal and oropharyngeal 3 | Q048 |
| Date PCR result returned? | └─┴─┘ └─┴─┘ └─┴─┴─┴─┘  Day/Month/Year | Q049 |
| Name of PCR kit used |  | Q050 |
| Name of laboratory platform used |  | Q051 |
| Name of laboratory that did analysis |  | Q052 |

| 1. **Mode of contamination** | | |
| --- | --- | --- |
| **Question** | **Response** | **Code** |
| What type of space ? | Enclosed 1  Open 2  Closed ventilated 3  Other 4  If other, specify :………………………………………. | Q053 |
| Visited a patient with COVID-19 ? | Yes 1  No 2  Don’t know/ no answer NK | Q054 |
| Work with people infected with COVID-19 ? | Yes 1  No 2  Don’t know/ no answer NK | Q055 |
| Face-to-face contact with a COVID-19 patient at less than 1m distance ? | Yes 1  No 2  Don’t know/ no answer NK | Q056 |
| Shared an enclosed space with a COVID-19 patient (including classroom or cleaning or attending a same event)? | Yes 1  No 2  Don’t know/ no answer NK | Q057 |
| Travel (car/bus/taxi/own vehicle/plane) with a COVID-19 patient ? | Yes 1  No 2  Don’t know/ no answer NK | Q058 |
| Delivered care directly to COVID-19 patients ? | Yes 1  No 2  Don’t know/ no answer NK | Q059 |

ID Participant : Site : |____| Participant : |____|____|____|

| 1. **Clinical Characteristics and course of disease** | | |
| --- | --- | --- |
| **General status and vital signs at admission/first visit (within 5 days of the date of diagnosis):** | | |
| **Question** | **Response** | **Code** |
| Clinical data collected with 5 days after diagnosis? | Yes 1  No 2  Don’t know NK | Q059bis  **If 2 or NK, go to Q060** |
| Date of data recording | └─┴─┘ └─┴─┘ └─┴─┴─┴─┘  Day/Month/Year | Q060bis |
| General status ? | Good 1  Quite good 2  Altered 3  Not recorded NK | Q061 |
| Glasgow Score? | └─┴─┘ | Q062 |
| Temperature? | └─┴─┘.└─┘ °C | Q063 |
| Blood pressure? | SBP └─┴─┴─┘mmHg  DBP └─┴─┴─┘mmHg | Q064 |
| Weight  ? | └─┴─┴─┘ Kg Measured ☐  Estimated ☐ | Q065 |
| Height ? | └─┴─┴─┘ cm | Q066 |
| Oxygen saturation (pulse oxymetry) ? | └─┴─┴─┘% | Q067 |
| Undernourishment ? | Yes 1  No 2  Not recorded NK | Q068 |
| Severe dehydration ? | Yes 1  No 2  Not recorded NK | Q069 |
| Sternal capillary refill time > 2 seconds ? | Yes 1  No 2  Not recorded NK | Q070 |
| **History of the disease (symptoms/clinical signs)** | | |
| **Question** | **Response** | **Code** |
| Date on symptom onset (date of onset of the first symptom) ? | └─┴─┘ └─┴─┘ └─┴─┴─┴─┘  Day/Month/Year  If patient asymptomatic : NA | Q060 |

ID Participant : Site : |____| Participant : |____|____|____|

| Fever (≥ 38°C) or history of fever ? | Yes 1  No 2  Not recorded NK | | Q071 | | | |
| --- | --- | --- | --- | --- | --- | --- |
| Asthenia/fatigue/uneasiness? | Yes 1  No 2  Not recorded NK | | Q072 | | | |
| Myalgia/aching muscles ? | Yes 1  No 2  Don’t know/ no answer NK | | Q073 | | | |
| Joint pain ? | Yes 1  No 2  Don’t know/ no answer NK | | | Q074 | |  |
| Headache ? | Yes 1  No 2  Not recorded NK | | | Q075 | |  |
| Shivering (feeling of cold) ? | | Yes 1  No 2  Don’t know/ no answer NK | | | Q076 | |
| Cough ? | | Yes 1  No 2  Not recorded NK | | | Q077 | |
| Dyspnea (breathlessness) ? | | Yes 1  No 2  Not recorded NK | | | Q078 | |
| Wheezing ? | | Yes 1  No 2  Don’t know/ no answer NK | | | Q079 | |
| Headcold ? | | Yes 1  No 2  Don’t know/ no answer NK | | | Q080 | |
| Epistaxis (nosebleed) ? | | Yes 1  No 2  Don’t know/ no answer NK | | | Q081 | |
| Sore throat ? | | Yes 1  No 2  Don’t know/ no answer NK | | | Q082 | |
| Nausea / vomiting ? | | Yes 1  No 2  Don’t know/ no answer NK | | | Q083 | |
| Abdominal pain ? | | Yes 1  No 2  Don’t know/ no answer NK | | | Q084 | |

ID Participant : Site : |____| Participant : |____|____|____|

| Diarrhea ? | Yes 1  No 2  Don’t know/ no answer NK | Q085 |
| --- | --- | --- |
| Conjunctivitis ? | Yes 1  No 2  Don’t know/ no answer NK | Q086 |
| Ageusia (loss of taste) ? | Yes 1  No 2  Don’t know/ no answer NK | Q087 |
| Anosmia (loss of smell) ? | Yes 1  No 2  Don’t know/ no answer NK | Q088 |
| Anorexia (loss of appetite) ? | Yes 1  No 2  Don’t know/ no answer NK | Q089 |
| Rash (cutaneous eruption) ? | Yes 1  No 2  Don’t know/ no answer NK | Q090 |
| Consciousness disorders ? | Yes 1  No 2  Don’t know/ no answer NK | Q091 |
| Convulsions ? | Yes 1  No 2  Don’t know/ no answer NK | Q092 |
| Acute respiratory distress syndrome ? | Yes 1  No 2  Not recorded NK | Q093 |
| Chest pain ? | Yes 1  No 2  Don’t know/ no answer NK | Q094 |
| Sepsis ? | Yes 1  No 2  Not recorded NK | Q095 |
| Septic shock ? | Yes 1  No 2  Not recorded NK | Q096 |
| Multiorgan failure ? | Yes 1  No 2  Not recorded NK | Q097 |
| Other signs (specify) ?  ……………………………………….. | Yes 1  No 2 | Q098 |

ID Participant : Site : |____| Participant : |____|____|____|

| 1. **Biological work-up at admission /first visit (within 5 days of diagnosis)** | | |
| --- | --- | --- |
| **Complete Blood Count** | | |
| Were biological results recorded within 5 days of diagnosis? | Yes 1  No 2  Don’t know NK | Q098bis  **If 2 or NK, go to Q120** |
| Complete blood count performed/available ? | Yes 1  No 2 | Q099  **If 2, go to Q108bis** |
| White blood cells | └─┴─┘.└─┴─┘.10^3^ cells/ml | Q100 |
| Total Lymphocytes | └─┴─┘.└─┴─┘.10^3^ cells /ml | Q101 |
| Neutrophils | └─┴─┘.└─┴─┘.10^3^ cells /ml | Q102 |
| Monocytes | └─┴─┘.└─┴─┘.10^3^ cells /ml | Q103 |
| Eosinophils | └─┴─┘.└─┴─┘.10^3^ cells /ml | Q104 |
| Basophils | └─┴─┘.└─┴─┘.10^3^ cells /ml | Q105 |
| Red blood cells | └─┴─┘.└─┴─┘.10^6^ cells /ml | Q106 |
| Hemoglobin | └─┴─┘.└─┘g/dl | Q107 |
| Platelets | └─┴─┴─┘.10^3^ cells /ml | Q108 |
| **Biochemistry** | | |
| Biochemistry performed/available ? | Yes 1  No 2 | Q108bis  **If 2, go to Q120** |
| Glycemia | └─┴─┘.└─┘mmol/l | Q109 |
| Creatininemia | └─┴─┴─┴─┘mmol/µl | Q110 |
| ASAT | └─┴─┘.└─┘IU/l | Q111 |
| ALAT | └─┴─┘.└─┘IU/l | Q112 |
| Prothrombin rate | └─┴─┘% | Q113 |
| Total bilirubin | └─┴─┴─┴─┘mg/l | Q114 |
| Kalemia | └─┴─┘.└─┘mmol/l | Q115 |

ID Participant : Site : |____| Participant : |____|____|____|

| Calcemia | └─┴─┘.└─┘mmol/l | Q116 |
| --- | --- | --- |
| Magnesium | └─┴─┘.└─┘mmol/l | Q117 |
| D-Dimers | └─┴─┴─┴─┘µg/l | Q118 |
| C-Reactive Protein (CRP) | └─┴─┴─┴─┘mg/l | Q119 |

| 1. **Imaging at admission/first visit (within 5 days of diagnosis)** | | |
| --- | --- | --- |
| **Question** | **Response** | **Code** |
| Lung X-ray ? | Yes 1  No 2 | Q120 |
| If yes, | | |
| Lung X-ray findings |  | Q121 |
| Chest CT scan ? | Yes 1  No 2 | Q122 |
| If yes, | | |
| Chest CT scan findings |  | Q123 |
| Electrocardiogram? | Yes 1  No 2 | Q124 |
| If yes, | | |
| Electrocardiogram findings |  | Q125 |

| 1. **Respiratory support at admission/first visit (within 5 days of diagnosis)** | | |
| --- | --- | --- |
| Oxygen therapy? | Yes 1  No 2  Not recorded NK | Q126 |
| Non-invasive ventilation mask? | Yes 1  No 2  Not recorded NK | Q127 |
| Endotracheal intubation (invasive ventilation) ? | Yes 1  No 2  Not recorded NK | Q128 |

ID Participant : Site : |____| Participant : |____|____|____|

**Multidisciplinary Study of COVID-19 in Burkina Faso (EMuL-COVID-19), ANRS-COV13 : Clinical Epidemiology**

**Visits during hospital stay or follow-up**

| 1. **Clinical Events during hospital stay / follow-up** | | | | | | |
| --- | --- | --- | --- | --- | --- | --- |
| Please choose yes or no, and if yes, indicate date of first occurrence. | | | | | | |
|  | **Yes** | **No** | **Start date** | **Ongoing** | **End date** | **Code** |
| Fever (Temperature ≥ 38°C)? |  |  |  | ☐ |  | Q129 |
| Asthenia/fatigue/uneasiness? |  |  |  | ☐ |  | Q130 |
| Headache? |  |  |  | ☐ |  | Q131 |
| Cough? |  |  |  | ☐ |  | Q132 |
| Dyspnea (breathlessness)? |  |  |  | ☐ |  | Q133 |
| Wheezing |  |  |  | ☐ |  | Q134 |
| Pneumonia? |  |  |  | ☐ |  | Q135 |
| Severe pneumonia |  |  |  | ☐ |  | Q136 |
| Sore throat? |  |  |  | ☐ |  | Q137 |
| Runny nose? |  |  |  |  |  | Q138 |
| Conjunctivitis? |  |  |  | ☐ |  | Q139 |
| Acute bronchitis? |  |  |  | ☐ |  | Q140 |
| Nausea / vomiting ? |  |  |  | ☐ |  | Q141 |
| Abdominal pain ? |  |  |  | ☐ |  | Q142 |
| Diarrhea |  |  |  | ☐ |  | Q143 |
| Anosmia (loss of smell) ? |  |  |  | ☐ |  | Q144 |
| Ageusia (loss of taste) ? |  |  |  | ☐ |  | Q145 |
| Anorexia (loss of appetite) ? |  |  |  | ☐ |  | Q146 |
| Epistaxis ? |  |  |  | ☐ |  | Q147 |

ID Participant : Site : |____| Participant : |____|____|____|

|  | **Yes** | **No** | **Start Date** | **Ongoing** | **End Date** | **Code** |
| --- | --- | --- | --- | --- | --- | --- |
| Rash (cutaneous eruption) ? |  |  |  | ☐ |  | Q148 |
| Convulsions ? |  |  |  | ☐ |  | Q149 |
| Arthralgia ? |  |  |  | ☐ |  | Q150 |
| Myalgia/muscle pain ? |  |  |  | ☐ |  | Q151 |
| Respiratory distress syndrome ? |  |  |  | ☐ |  | Q152 |
| Chest pain ? |  |  |  | ☐ |  | Q153 |
| Coagulation disorders ? |  |  |  | ☐ |  | Q154 |
| Sepsis ? |  |  |  | ☐ |  | Q155 |
| Septic shock ? |  |  |  | ☐ |  | Q156 |
| Multiorgan failure ? |  |  |  | ☐ |  | Q157 |
| Dialysis ? |  |  |  | ☐ |  | Q158 |
| Coma ? |  |  |  | ☐ |  | Q159 |
| Other sign (specify) ?  ……………………………………………. |  |  |  | ☐ |  | Q160 |

ID Participant : Site : |____| Participant : |____|____|____|

| 1. **Biology/Electrolyte Disorders occurring during hospital stay / follow-up** | | | | | | |
| --- | --- | --- | --- | --- | --- | --- |
| Please choose yes or no, and if yes, indicate date of first occurrence | | | | | | |
|  | **Yes** | **No** | **Start Date** | **Ongoing** | **End Date** | **Code** |
| Hyperleukocytosis |  |  |  | ☐ |  | Q161 |
| Lymphopenia |  |  |  | ☐ |  | Q162 |
| Anemia |  |  |  | ☐ |  | Q163 |
| Thrombopenia |  |  |  | ☐ |  | Q164 |
| Elevated transaminases |  |  |  | ☐ |  | Q165 |
| Elevated bilirubin |  |  |  | ☐ |  | Q166 |
| Hyperkalemia |  |  |  | ☐ |  | Q167 |
| Hypokalemia |  |  |  | ☐ |  | Q168 |
| Hypercalcemia |  |  |  | ☐ |  | Q169 |
| Hypocalcemia |  |  |  | ☐ |  | Q170 |
| Hypermagnesemia |  |  |  | ☐ |  | Q171 |
| Hypomagnesemia |  |  |  | ☐ |  | Q172 |
| Hypercreatininemia |  |  |  | ☐ |  | Q173 |
| Elevated D-Dimers |  |  |  | ☐ |  | Q174 |

| 1. **Abnormal imaging findings during hospital stay / follow-up** | | | | | | |
| --- | --- | --- | --- | --- | --- | --- |
| Please choose yes or no, and if yes, indicate date of first occurrence | | | | | | |
|  | **Yes** | **No** | **Start Date** | **Ongoing** | **End Date** | **Code** |
| Cardiac arrhythmia |  |  |  | ☐ |  | Q175 |
| Pulmonary parenchyma lesions |  |  |  | ☐ |  | Q176 |
| Alveolar lesions |  |  |  | ☐ |  | Q177 |
| Pleural lesions |  |  |  | ☐ |  | Q178 |

ID Participant: Site : |____| Participant : |____|____|____|

| 1. **Respiratory support during hospital stay / follow-up** | | | | | | |
| --- | --- | --- | --- | --- | --- | --- |
| Please choose yes or no, and if yes, indicate date of first occurrence | | | | | | |
|  | **Yes** | **No** | **Start Date** | **Ongoing** | **End Date** | **Code** |
| Oxygen therapy |  |  |  | ☐ |  | Q179 |
| Endotracheal intubation (invasive ventilation) |  |  |  | ☐ |  | Q180 |
| Non-invasive ventilation mask |  |  |  | ☐ |  | Q181 |

| 1. **Follow-up of PCR results** | | | | | |
| --- | --- | --- | --- | --- | --- |
| **Date of sample**  Day/Month/Year | **Result** | | | **Name of laboratory** | **Code** |
|  | **Positive** | **Negative** | **Undetermined** |  | Q182 |
| └─┴─┘ └─┴─┘ └─┴─┴─┴─┘ |  |  |  |  |  |
| └─┴─┘ └─┴─┘ └─┴─┴─┴─┘ |  |  |  |  |  |
| └─┴─┘ └─┴─┘ └─┴─┴─┴─┘ |  |  |  |  |  |
| └─┴─┘ └─┴─┘ └─┴─┴─┴─┘ |  |  |  |  |  |
| └─┴─┘ └─┴─┘ └─┴─┴─┴─┘ |  |  |  |  |  |
| └─┴─┘ └─┴─┘ └─┴─┴─┴─┘ |  |  |  |  |  |

**Discharge**

| 1. **Mode of Discharge** | | | | | |
| --- | --- | --- | --- | --- | --- |
| Tick the appropriate box | | | | | |
|  | | **Yes** | **No** | **Comments** | **Code** |
| Recovered without sequelae | |  |  |  | Q183 |
| Recovered with sequelae (specify type of sequelae) | |  |  |  |  |
| Discharge against medical advice | |  |  |  |  |
| Referred to another healthcare establishment | |  |  |  |  |
| Out of isolation, not recovered | |  |  |  |  |
| Deceased | |  |  |  |  |
| Date of discharge/death/referral└─┴─┘ └─┴─┘ └─┴─┴─┴─┘  Day/Month/Year | | | | | Q184 |
| If patient deceased, specify the cause of death (WHO classification)? | Disease 1  Accident 2  Intentional self-harm 3  Assault 4  Legal intervention 5  War 6  Could not be determined 7  Pending investigation 8  Unknown 9 | | | | Q185 |
| Did the patient receive treatment ? | Yes 1  No 2  Don’t know / Not recorded NK | | | | Q186  **If 1, complete the treatment form** |

ID Participant: Site : |____| Participant : |____|____|____|

| **Treatment Form** | | | | | |
| --- | --- | --- | --- | --- | --- |
| **N°** | **Name of molecule (INN)** | **Start Date** | **Ongoing** | **End Date** | **Reason for discontinuation** |
| 1 |  |  | ☐ |  |  |
| 2 |  |  | ☐ |  |  |
| 3 |  |  | ☐ |  |  |
| 4 |  |  | ☐ |  |  |
| 5 |  |  | ☐ |  |  |
| 6 |  |  | ☐ |  |  |
| 7 |  |  | ☐ |  |  |
| 8 |  |  | ☐ |  |  |
| 9 |  |  | ☐ |  |  |
| 10 |  |  | ☐ |  |  |
| 11 |  |  | ☐ |  |  |
| 12 |  |  | ☐ |  |  |
| 13 |  |  | ☐ |  |  |
