# Supplementary material for: COVID-19 epidemiological, sociological and anthropological investigation: study protocol for a multidisciplinary mixed methods research in Burkina Faso
Source: BMC Infect Dis. 2021 Sep 3;21:896. doi: 10.1186/s12879-021-06543-4 (PMC8414025; doi:10.1186/s12879-021-06543-4)
Supplement: Supplementary file 3 — Additional file 3. English translation of the qualitative interview guide (Phase 3): general population. [file 12879_2021_6543_MOESM3_ESM.docx]

**Additional file 3a: English translation of the qualitative interview guide: general population**

**Multidisciplinary Study of COVID-19 in Burkina Faso (EMuL-COVID-19), ANRS-COV13: Socio-anthropological study**

**Interview guide to explore perceptions, acceptability, and applicability of preventive measures against COVID-19, general population**

1. **Introduction**

Hello. My name is ________________________ I have been sent by the MURAZ Centre to do a study about the coronavirus (COVID-19). The main aim of this study is to investigate the healthcare pathways and experiences of patients who attend COVID-19 care reference, and to explore the perceptions, acceptability, and applicability of preventive strategies against infection among healthcare workers and the general population. The information you choose to give us will remain strictly confidential. The interview will last about **45 minutes**. With your permission, we are going to record the interview, to ensure that we accurately transcribe what you said. Thank you in advance for your participation.

1. **Detailed Discussion**

| **Perception of COVID-19** |
| --- |

1. Knowledge about COVID-19

- Definition: what is the local name/How do you call it in your local languages? What does it mean? Where is the origin?
- Transmission – contagion: describe modes of transmission – contagion?
- Signs and symptoms: what are the signs/symptoms of COVID-19?
- Causes of COVID-19: what are the causes of COVID-19 (disease from God, witchcraft, fight for world domination etc.)
- Perceived risk: Do you think you are at risk of COVID-19? Why?
- Care: What kind of care exists (healthcare, traditional medicine, other) to treat COVID-19? Efficacy and challenges of each type of care.

1. Prevention: What are the advantages, difficulties, and disadvantages / acceptability / applicability

- Official preventive measures / methods: What are the different official preventive measures? (Availability, cost, use, efficacy)
- Use of non-official preventive measures (traditional, other): availability, cost, use, efficacy
- Advantages: What does effective application of the different preventive measures (by the method mentioned by the respondent) achieve?
- Difficulties: what makes the application of the different preventive methods difficult? (specifically, the method mentioned by the respondent)
- Disadvantages: What aspects of daily life are lost by effective application of the different preventive measures (specifically the method mentioned by the respondent)
- Changes to your social life: family, friends, professional life, associations, community

| Acceptability of the preventive measures/ care procedures |
| --- |

- Acceptability:
  - Among the methods/measures mentioned earlier, which of them can be respected / accepted in our context? Which ones cannot? Why?
  - Among the methods/measures mentioned earlier, which of them do you want to respect / accept in our context? Which ones do you not respect/accept? Why?

| Applicability |
| --- |

- Applicability:
  - Among the methods/measures mentioned earlier, which of them do you implement? (Which ones do you put into practice easily? Which ones are difficult to implement and why? Which ones are you unable to implement and why?)
  - What suggestions would you like to make for improved acceptability / applicability of these methods/measures in our context?
- **Is there anything else you would like to add?**

**Additional file 3b: English translation of the qualitative interview guide: healthcare professionals**

**Multidisciplinary Study of COVID-19 in Burkina Faso (EMuL-COVID-19), ANRS-COV13: Socio-anthropological study**

**Interview guide to explore the perceptions of healthcare professionals**

1. **Introduction**

Hello. My name is ________________________ I have been sent by the MURAZ Centre to do a study about the coronavirus (COVID-19). The main aim of this study is to investigate the healthcare pathways and experiences of patients who attend COVID-19 care reference, and to explore the perceptions, acceptability, and applicability of preventive strategies against infection among healthcare workers and the general population. The information you choose to give us will remain strictly confidential. The interview will last about **45 minutes**. With your permission, we are going to record the interview, to ensure that we accurately transcribe what you said. Thank you in advance for your participation.

1. **Detailed Discussion**

| **Organization of healthcare delivery** |
| --- |

1. Origin of patients with COVID-19: healthcare referrals, personal consultation, etc.
2. Availability of biomedical equipment: gloves, masks, hygiene (existence or absence of facilities for handwashing, disinfection etc).
3. Staff: your opinion regarding the quantity (number) and quality (level of training, competence for the management of the COVID-19 epidemic and patient management)
4. Conditions of healthcare delivery: availability of COVID-19 tests, acceptance, or refusal of tests by patients on arrival in the hospital, availability of treatments for COVID-19, appreciation of visits to patients (if visits were allowed).
5. Perception of the hospitalized patients by the healthcare staff: compliance with treatment administered in hospital, did you notice any parallel treatments, relationship between healthcare workers and patients.
6. Risks: sufficient protection or exposure to contamination by COVID-19 during care delivery, perception among those in your social environment (family, friends).
7. What is your opinion on the preventive methods: list the different methods, accessibility...?
8. Difficulties encountered in delivering healthcare in general.

- **Is there anything else you would like to add?**

**Additional file 3c: English translation of the qualitative interview guide: patients treated in health care centers dedicated to COVID-19 patients**

**Multidisciplinary Study of COVID-19 in Burkina Faso (EMuL-COVID-19), ANRS-COV13: Socio-anthropological study**

**Interview guide to explore the experiences of patients managed in healthcare centers**

1. **Introduction**

Hello. My name is ________________________ I have been sent by the MURAZ Centre to do a study about the coronavirus (COVID-19). The main aim of this study is to investigate the healthcare pathways and experiences of patients who attend COVID-19 care reference, and to explore the perceptions, acceptability, and applicability of preventive strategies against infection among healthcare workers and the general population. The information you choose to give us will remain strictly confidential. The interview will last about **45 minutes**. With your permission, we are going to record the interview, to ensure that we accurately transcribe what you said. Thank you in advance for your participation.

1. **Detailed Discussion**

| **Experience of the disease in COVID-19 patients** |
| --- |

1. **Experience of the disease before hospital admission**

- Test and reasons for test: what prompted you to get tested for COVID-19?
- Sample swab: Can you tell us about the test sampling (when was it? What did you think of it, any difficulties encountered)?
- Announcement of the results: What was your experience of receiving the test results (duration, the shock, any psychological support or not)
- Sharing the results, reaction of your entourage: Who did you tell about your result? (Support, rejection, stigmatization, etc).
- Isolation in your home: What protective measures did you have to implement? Any difficulties encountered?
- (Self-)medication before treatment initiation: did you take any treatments before the initiation of treatment in the hospital (self-medication, traditional medicine, modern medicine, traditional-practitioners, medical staff, healthcare groups etc)
- Perception of COVID-19 during this period: What is your opinion about COVID-19?
- Any changes in your lifestyle: what changes (positive or negative) have occurred in your life?
- Any suggestions for improved screening?

1. **Experience of the disease during the hospital stay**

- Treatment received: Can you tell me about the treatments you received in the healthcare centre? (Type of treatment: tablets, infusion, blood draws, side effects, any psychological support received)
- Any other treatments during and after management in the healthcare centre: what other treatment strategies did you implement (other than those recommended by the healthcare centre) during and after your hospital stay?
- Relationship with the healthcare workers: can you tell us about the relations with the different healthcare workers throughout your experience in the centre (welcome, availability, communication, behaviour: recount your experience)
- Organization of management: What is your opinion about the organization of management in the healthcare centre (hygiene in the centre, spacious or not, food, isolation of patients, opening hours for services, visits to patients: recount your experience).
- Access to treatment: can you tell us about the access to treatment (free or paying, payment required for certain services etc).
- Changes in lifestyle: what changes (positive or negative) has COVID-19 brought about in your life?
- Any suggestions for improved management: What suggestions would you like to make to improve the management of patients with COVID-19 in the healthcare centres?

1. **Experience after discharge from hospital**

- Mode of discharge from hospital: were you discharged by the administration of the hospital, or did you leave of your own accord? If so, why? (Treatment not efficacious, unable to pay the costs, etc.)
- What is your state of health now? Recovered and in good health; recovered with sequelae; describe symptoms related to COVID-19.

1. **Is there anything else you would like to add?**
